# Metabolic Profiling of the EmDia Cohort by LC‐MS Reveals Empagliflozin‐Intake Associated Regulation of 1,5‐anhydroglucitol and Urate

**DOI:** 10.1002/pmic.70075

**Published:** 2025-12-03

**Authors:** Fabian Schmitt, Vincent ten Cate, Zlatka Fischer, Mathias Hagen, Barbara A. Steigenberger, Stefan Tenzer, Philipp S. Wild, Thierry Schmidlin

**Affiliations:** ^1^ Institute of Immunology University Medical Center Johannes Gutenberg University Mainz Mainz Germany; ^2^ Preventive Cardiology and Preventive Medicine Department of Cardiology University Medical Center of the Johannes Gutenberg University Mainz Mainz Germany; ^3^ Clinical Epidemiology and Systems Medicine Center For Thrombosis and Hemostasis (CTH) Mainz Germany; ^4^ German Center for Cardiovascular Research (DZHK) Partner Site Rhine‐Main Mainz Germany; ^5^ Mass Spectrometry Core Facility Max Planck Institute of Biochemistry Martinsried Germany; ^6^ Research Center for Immunotherapy (FZI) University Medical Center of the Johannes‐Gutenberg University Mainz Germany; ^7^ Institute For Molecular Biology (IMB) Mainz Germany

**Keywords:** data‐independent acquisition, human plasma, metabolomics, SGLT2 inhibitor, type 2 diabetes mellitus

## Abstract

The EmDia trial, designed to study the effects of the sodium glucose cotransporter‐2 (SGLT2) inhibitor empagliflozin on cardiovascular comorbidities in type 2 diabetes mellitus (T2DM) patients, has been investigated for short‐term metabolic alterations by a limited set of clinical assays. To expand on this data, we report on the development of a liquid chromatography‐mass spectrometry (LC‐MS)‐based metabolomics approach employing an optimized metabolite separation by pentafluorophenyl chromatography. High‐confidence metabolite annotation based on reference standards allows for fast and robust metabolic characterization of large plasma cohorts due to scalability. Applied to EmDia, we show the high predictive power of our methodology for several clinical parameters, including a near‐perfect prediction of fasting blood glucose (*R^2^* = 0.97), and demonstrate how empagliflozin leads to reduced plasma levels of deoxyhexoses, such as 1,5‐anhydroglucitol, a short‐term biomarker for glycemic control.

**Summary:**

- Clinical metabolomics studies continue to gain interest due to their comprehensive metabolite coverage, offering insights into metabolic alterations in health and disease.
- In this study, we present a robust data‐independent acquisition liquid chromatography‐mass spectrometry‐based metabolomics workflow employing an optimized metabolite separation by pentafluorophenyl chromatography that showcases a comprehensive coverage of plasma metabolites.
- Applied to characterize plasma metabolite profiles in samples of EmDia, a placebo controlled study investigating the effect of the SGLT2 inhibitor empagliflozin, we assess the predictive power of metabolite signals for clinical parameters describing organ physiologies and pathophysiologies.
- Descriptive statistics are applied to the metabolite profiles to identify empagliflozin intake‐associated metabolite markers.

## 1 Introduction

Diabetes mellitus affects more than 500 million people worldwide. Type 2 diabetes mellitus (T2DM) makes up over 95% of all diabetes cases, and we face an ever‐increasing prevalence, primarily caused by increasing obesity rates [1]. It is expected that T2DM will remain a major global epidemical burden [2] and T2DM patients often suffer from comorbidities, most prominently cardiovascular disease (CVD), [3] being a leading cause of morbidity and mortality in patients with T2DM [4]. Therefore, the T2DM medication empagliflozin, a sodium glucose cotransporter‐2 (SGLT2) inhibitor, is currently investigated for its potential to improve outcomes for comorbidities, for example, in the EmDia study, a randomized, double‐blind, and placebo‐controlled clinical study, aiming to evaluate the short‐term effects of empagliflozin on left ventricular diastolic function [5]. Empagliflozin has already showed to cause a reduction in cardiovascular events, heart failure‐related hospitalizations, and mortality in the EMPA‐REG OUTCOME study [6]. The EmDia trial confirmed that empagliflozin also improved left ventricular diastolic function, including in patients with heart failure with preserved ejection fraction, a patient subgroup with poor response to many treatments [7]. Next to its cardioprotective effects, empagliflozin reduces renal absorption and increases urinary excretion of glucose in an insulin‐independent manner. This results in decreased fasting plasma glucose (FPG) and hemoglobin A1c (HbA1c) levels and generally improved glycemic control, implying systemic drug‐related metabolic alterations [8, 9]. Metabolic changes in patient groups are commonly monitored using clinical tests. Yet, they are typically limited to certain specific metabolic markers, such as FPG and uric acid. In contrast, the application of liquid chromatography‐mass spectrometry (LC‐MS)‐based metabolomics provides a more comprehensive approach for profiling of metabolites from patient‐derived samples and has therefore several promising applications such as (1) identification of potentially novel diagnostic or prognostic biomarkers, (2) prediction of disease systems, and (3) representation of different organ systems in the body, in health and disease.

However, to date, the LC‐MS metabolomics toolbox encompasses a wide variety of methodological approaches differing substantially in metabolite coverage, confidence, quantitative accuracy, and capacity for throughput. For example, targeted metabolomics using isotopically labeled internal standards allows for the absolute quantification of a predefined set of metabolites. The obvious caveat being limitations in metabolite coverage due to availability and high costs of labeled standards [10]. Contrary to that, untargeted metabolomics takes into account detectable signals of all metabolites within a sample and can offer broad, unbiased relative quantification of metabolic signals, regardless of whether they are annotated or not [11]. Confidence levels obtained from untargeted metabolomics analysis can thus range from mere MS feature detection up to more confident metabolite annotation using MS/MS spectral matching with well‐curated spectral libraries.

Here, we describe a scalable fit‐for‐purpose data‐independent acquisition (DIA) LC‐MS workflow for metabolic profiling of the 432 patient plasma samples of the EmDia cohort. Our workflow is characterized by an easy‐to‐implement sample preparation protocol, high quantitative reproducibility, reliable metabolite annotation based on spectral libraries obtained from reference compounds, low computational demand during data analysis, and a throughput of up to 120 sample measurements per day. We used this approach to profile > 170 metabolites in the EmDia cohort.

## 2 Results

### 2.1 EmDia Study Design, Sample Preparation, and Metabolomics Analysis

**FIGURE 1:**
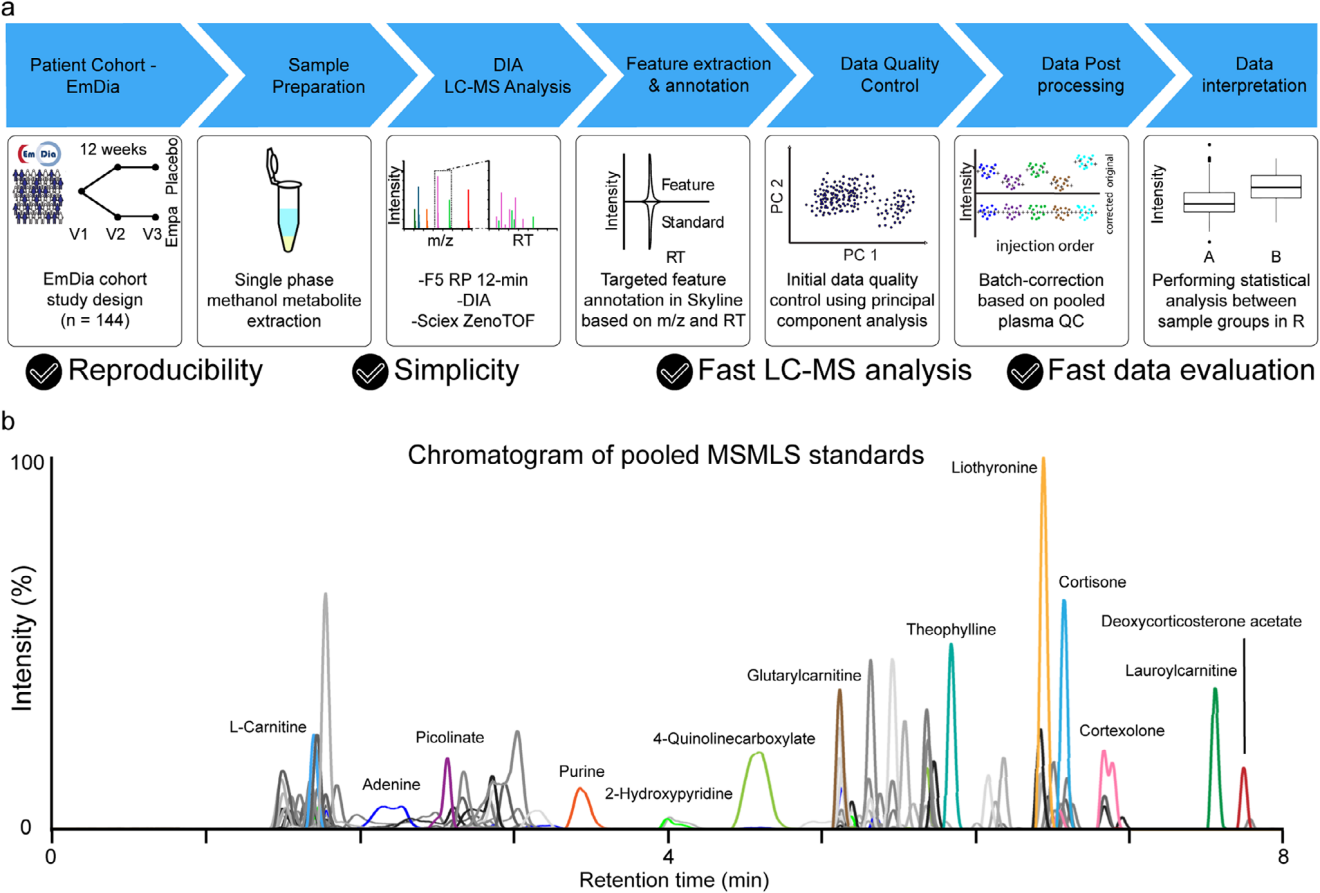
Metabolomics analysis of EmDia cohort: (a) Workflow used for metabolic profiling of plasma samples from the EmDia cohort. Ethylenediaminetetraacetic acid (EDTA) plasma samples were included for all 144 patients enrolled in the EmDia study at three time points, prior to treatment (V1), after 1 week (V2) and after 12 weeks (V3). Metabolites were extracted from plasma using methanol extraction protocol. All samples were analyzed using LC‐MS, employing pentafluorophenyl (F5) chromatography for metabolite separation followed by data‐independent acquisition. Metabolites were annotated across all samples using targeted feature annotation based on our in‐house spectral library containing explicit retention time (RT) and molecular formula. Principal component analysis was performed to assess batch effects, which were corrected for by applying the quality control‐robust spline correction (QC‐RSC). Statistical analysis to assess group‐specific differences in metabolite abundance was performed in *R*. (b) Exemplary chromatogram of pooled reference compounds of the mass spectrometry metabolite library of standards (MSMLS) acquired using separation by a F5 column. XICs of selected metabolites from different chemical subclasses are highlighted for illustrative purposes.

The placebo‐controlled EmDia study was designed to investigate the effect of empagliflozin in patients with T2DM and elevated left diastolic pressure. 144 patients were split 1:1 in placebo and empagliflozin group, and blood was collected from all patients prior to the first drug administration (baseline, V1), after 1 week (V2), and after 12 weeks (V3) [5]. For metabolic profiling of the EmDia cohort, we set out to establish a DIA‐LC‐MS‐based metabolomics workflow, characterized by a high analytical reproducibility (median CV values typically 6%–10% prior to batch correction and ≤ 6% after batch correction (Figures S1 and S2), and the capacity for high‐throughput of up to 120 injections per day, due to a relatively short analysis time of 12 min (Figure 1).

### 2.2 Pentafluorophenyl Chromatography Provides Broad Metabolite Coverage

We generated a carefully curated in‐house spectral library based on the 612 synthetic standard compounds of the mass spectrometry metabolite library of standards (MSMLS) used as reference for metabolite annotation [12]. In order to achieve the high‐throughput and robustness necessary for large plasma cohort analysis, while maintaining a comprehensive coverage of metabolites, we relied on reversed phase (RP) chromatography using a pentafluorophenyl (F5) column instead of hydrophilic interaction liquid chromatography (HILIC). While HILIC can achieve baseline separation of isobaric sugars, it involves long analysis times due to prolonged equilibration and requires careful maintenance of pH and mobile phase additives to avoid retention time (RT) shifts [13, 14]. The F5 column demonstrated retention of 487 compounds, covering a broad range of metabolite classes such as amino acids and analogues, organic acids, nucleobases, steroids, bile acids, fatty acids, and fatty acid derivatives (Figure 1b and Figure S3a). No obvious bias for specific compound classes was observed. Overall, sharp and symmetric chromatographic peak shapes were observed for most of the standard compounds, and several pairs of isobaric metabolites could be baseline separated, including leucine/isoleucine, betaine/valine, and theophylline/theobromine (Figure S4).

**FIGURE 2:**
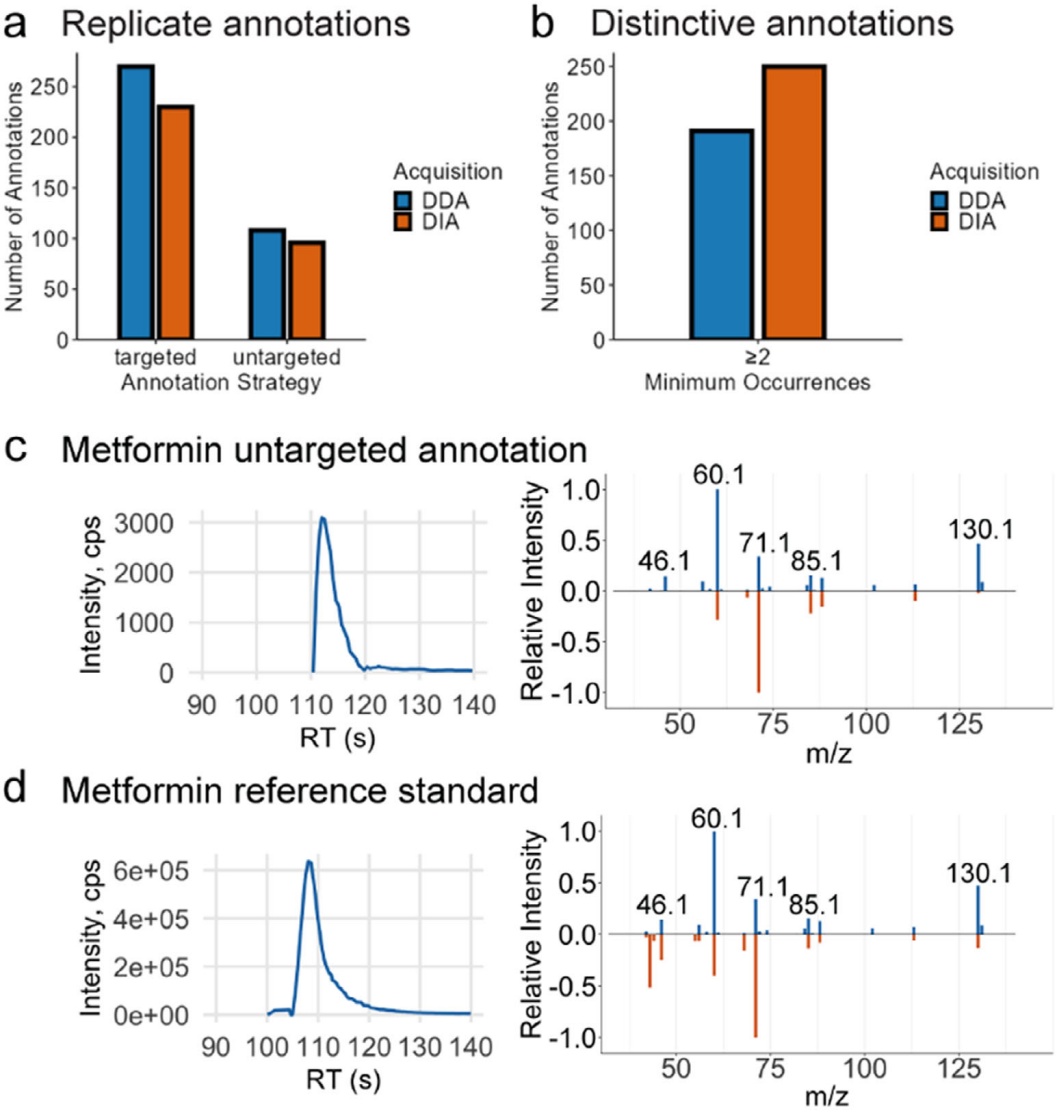
Metabolite annotation in DIA and DDA and Library Expansion. (a) Bar plot showing the number of metabolite annotations observed in 5 out of 5 SRM 1950 metabolite extraction replicates (replicate annotations) obtained by targeted (left) and untargeted (right) metabolite annotation, respectively for MS acquisition by DIA and DDA. (b) Bar plot showing the number of metabolite annotations obtained in a minimum of 2 out of 5 SRM 1950 metabolite extraction replicates (distinctive annotations) for DIA and DDA, respectively, using untargeted metabolite annotation. (c) XIC of metformin measured in pooled plasma QC samples of the EmDia cohort (left). Butterfly plot of fragmentation spectra showing deconvoluted DIA fragment spectrum of metformin measured in pooled QC (blue) contrasted to reference spectrum deposited in the NIST 2017 spectral library (orange) (right). (d) XIC of metformin synthetic standard (left). Butterfly plot of fragmentation spectra showing deconvoluted DIA fragment spectrum of metformin measured in pooled QC (blue) contrasted to the fragment spectrum of metformin reference acquired in‐house (orange).

Benchmarking our spectral library with a NIST SRM 1950 (SRM 1950) plasma extract analyzed in data‐dependent acquisition (DDA), we were able to reproducibly annotate 270 metabolites, when considering adducts as separate annotations, which predominantly included amino acids and analogues, purine derivatives, steroids, and fatty acids (Figure 2a and Figure S3b). Compound‐specific RTs observed in the plasma sample consistently matched RTs recorded in the measurements of pure MSMLS standards (Δ_RTmax_ = 0.1 min) (Figure S4). No chromatographic separation was achieved for isobaric sugars; hence, we here report them in the form of generic names based on their molecular formula.

### 2.3 Synergizing Targeted and Untargeted Metabolite Annotation for Comprehensive Metabolomics Data Analysis

We further evaluated the use of DDA and DIA acquisition methods. To that end, we acquired five injection replicates of SRM 1950 plasma in DDA and five replicates in DIA. We analyzed these 2 × 5 runs to benchmark targeted and untargeted data analysis. We initially focused on annotation reproducibility across all five injection replicates. Targeted analysis relied on feature detection with subsequent annotation exclusively based on known precursor masses and RT values of MSMLS compounds, and showed 17% higher numbers in DDA than DIA with 270 and 230 replicate annotations, respectively, when considering adducts separately (Figure 2a). We contrasted this analysis strategy to an untargeted analysis where metabolite annotation is exclusively based on MS/MS spectral similarity to the entries of the NIST 2017 spectral library. With this we could show that DDA outperformed DIA in this analysis with 108 annotations compared to 96, respectively. Yet, untargeted analysis of both DDA and DIA did not reach the metabolic depth we were able to achieve through targeted analysis (Figure 2a).

In addition, we evaluated distinctive annotations by looking into the total number of metabolite annotations, only filtering based on score and a much less stringent requirement of annotations being present in at least two replicates to avoid inclusion of too many false positives. In this analysis, the highest number of total metabolite annotations was found using DIA data with 250 annotations, resulting in roughly 31% more distinctive annotations compared to the 191 distinctive annotations in DDA data (Figure 2b). We observed an overlap of 101 annotations matched by compound name (Figure S5). Untargeted analysis of DIA showed higher numbers of annotations than DDA for distinctive annotations occurring in two out of five, three out of five, and four out of five (Figure S6) and exhibited 15% higher metabolite annotations across samples with an average of 249.6 annotations in DDA and 216 in DIA (Figure S7).

When analyzing plasma samples of the EmDia cohort and their corresponding pooled QC samples, we relied on DIA acquisition combined with MS1‐based manual targeted feature annotation in Skyline. Yet, we decided to expand the target list to include selected additional compounds not present in the MSMLS, which had been annotated during untargeted data analysis QC data. To ensure confidence in these additional annotations, we dynamically expanded the in‐house spectral library by acquiring reference data from synthetic standards of these compounds, such as metformin (Figures 2c,d), empagliflozin, glycerophosphocholine, N‐methylarginine, and N‐methyl‐2‐pyridone‐5‐carboxamide.

### 2.4 Metabolite Profiles Show High Predictive Values for Glycemic Control, Kidney, and Liver (Patho‐)Physiology

We applied the methodology described to metabolically phenotype all 432 samples of the EmDia cohort. We confidently annotated 175 metabolites, 135 in positive ion mode and 120 in negative ion mode (Table S1). High empagliflozin levels were exclusively observed in the plasma of patients in the empagliflozin group in V2 and V3, while they stayed around noise level at V1 and the placebo group (Figure S8).

**FIGURE 3:**
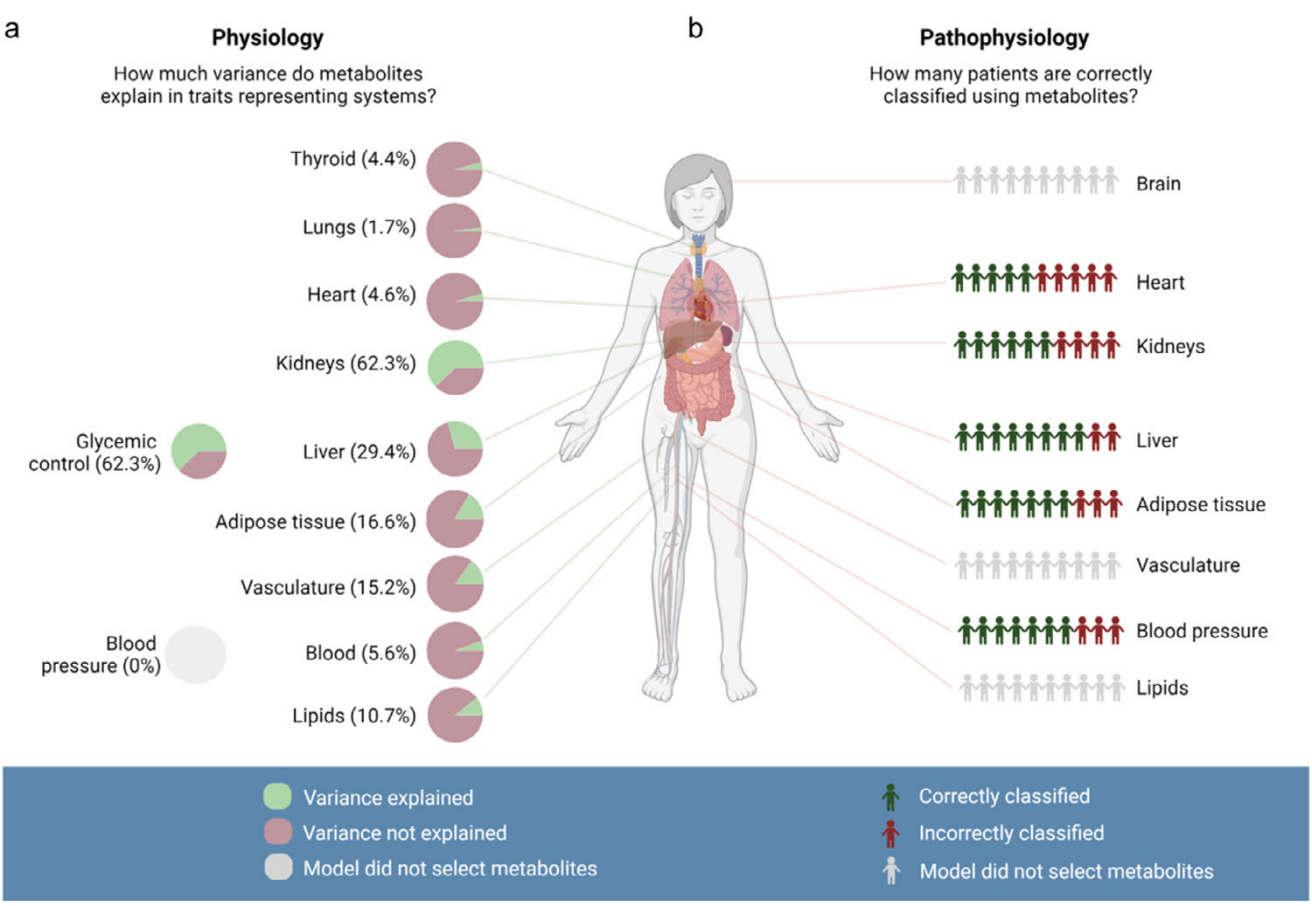
Assessment of predictive power of metabolites for clinical traits (see Table 1 for underlying clinical traits). (a) Percentage of variance explained by the metabolomics data indicated as a pie chart for clinical parameters or sets of clinical parameters reflective of organ physiology for 11 organs, as assessed by net‐regularized linear and logistic regression models for all patients at V1. Best prediction models were achieved for kidney physiology, reflected by eGFR, and glycemic control, reflected by glucose and HbA1c. Gray charts indicate organ physiology for which no metabolites were selected. (b) Suitability of the models to discriminate individuals with organ‐specific diseases, classified by the area under the receiver operating characteristic curve (AUC), was assessed for a set of diseases. Diseases where no metabolites were selected are indicated in gray.

We initially assessed the predictive power of the metabolomics data based on the metabolic profiles acquired for the 144 patients at V1. We used elastic net‐regularized linear and logistic regression models to identify metabolites associated with continuous clinical traits used to represent functionality of specific organs (Figure 3a, Table 1 and Tables S4–S8) as well as identifying metabolites capable of discriminating organ‐specific diseases based on known, commonly used diagnosis markers of the patients (Figure 3b, Table 1 and Tables S9–S13).

**TABLE 1:** Clinical values defining organ physiology and pathophysiology.

Organ system	Clinical traits
Adipose tissue	Body mass index Waist‐to‐height ratio
Blood	White blood cells Red blood cells Neutrophils Eosinophils Basophils platelet count Mean platelet volume Lymphocytes Monocytes International normalized ratio Activated partial thromboplastin clotting time
Blood pressure	Systolic blood pressure Diastolic blood pressure
Heart	NT‐proBNP Troponin I Left ventricular E/E' Left ventricular EF Left ventricular mass
Kidneys	eGFR
Lipids	Triglycerides Total cholesterol LDL cholesterol HDL cholesterol Apolipoprotein B Apolipoprotein A
Liver	Fatty liver index Fib4 liver fibrosis score Glutamic oxaloacetic transaminase Gamma‐glutamyl transferase Glutamic pyruvic transaminase
Lungs	Forced expiratory volume in one second Forced vital capacity
Glycemic Control	Glucose HbA1c
Thyroid	Thyroid‐stimulating hormone
Vasculature	Aortic pulse wave velocity Carotid‐femoral pulse wave velocity Aortic augmentation index Ankle‐brachial index Carotid intima‐media thickness

**Organ system**	**Disease classification**
Adipose tissue	Obesity
Blood pressure	Arterial hypertension
Brain	History of stroke
Heart	Atrial fibrillation History of myocardial infarction Congestive heart failure
Kidneys	Chronic kidney disease
Lipids	Dyslipidemia
Liver	Fatty liver disease Liver fibrosis
Vasculature	Coronary artery disease Peripheral artery disease

We found that our data showed the highest association to common clinical markers used to assess glycemic control (glucose, HbA1c) and kidney function (estimated glomerular filtration rate (eGFR)). The elastic net regression model extracted 34 metabolic markers of interest representing kidney function as determined by known clinical eGFR values for the patients. This resulted in an overall 10‐fold cross‐validated *R^2 ^* = 0.63, meaning that 63% of the variance in a marker representative of kidney function can be recapitulated by these metabolites. Creatinine signals measured in our metabolomics analysis in positive ion mode showed the highest contribution to the model, with a slightly lower, yet still high contribution when measured in negative ion mode. Additional metabolic signals contributing to the model included kynurenate, the amino acids guanidinosuccinate, N,N‐dimethylarginine, glutamate, isoleucine, leucine, and glutamine, as well as uric acid (Table S4).

**FIGURE 4:**
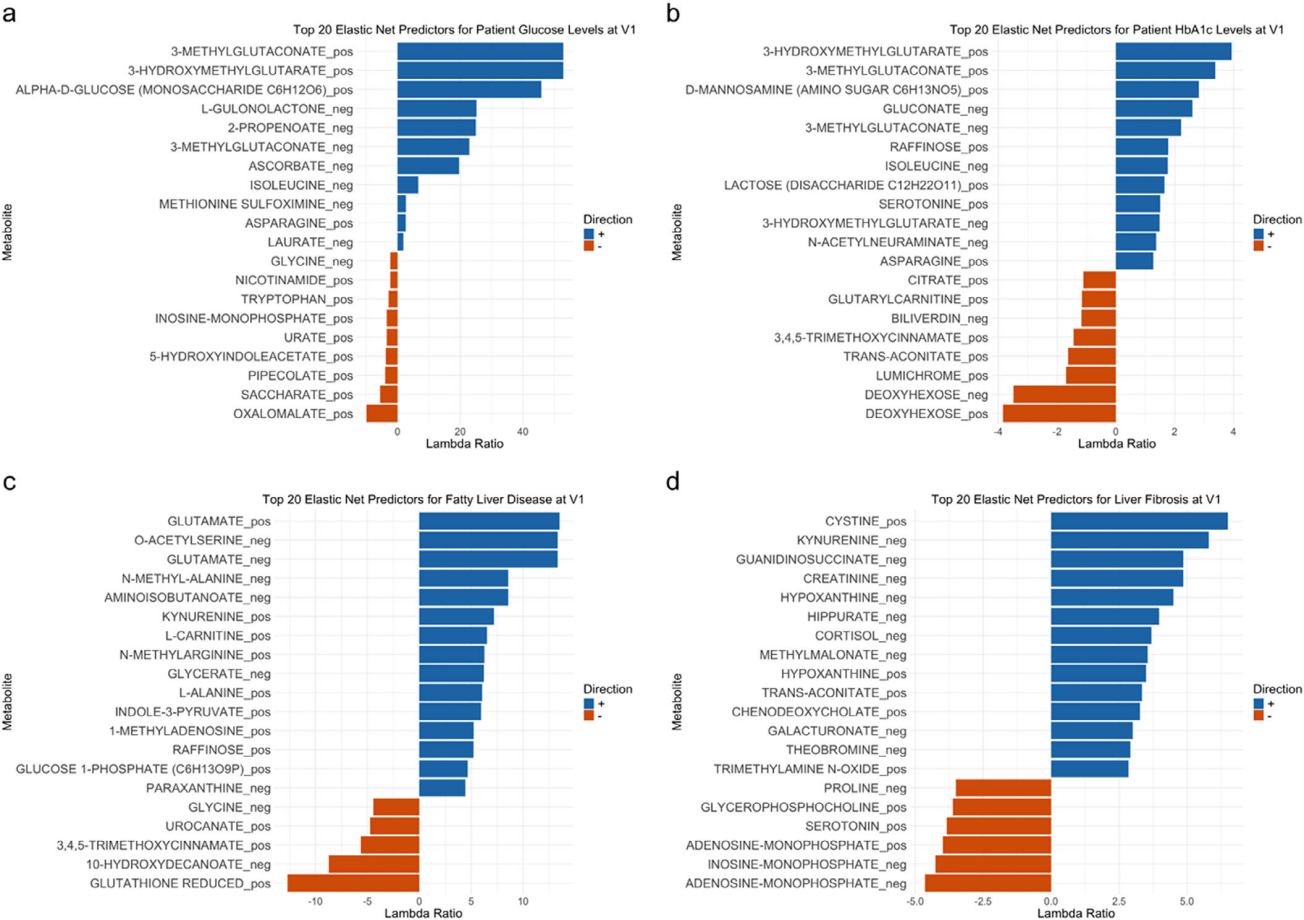
Lambda ratios of the top 20 predictors selected by elastic net regression for prediction models built to explain the clinical traits at V1 prior to empagliflozin or placebo treatment. (a) Top 20 predictors for glucose levels. (b) Top 20 predictors for HbA1c levels. (c) Top 20 predictors for fatty liver disease. (d) Top 20 predictors for liver fibrosis.

To represent glycemic control of individuals, clinically established levels of glucose and HbA1c were used. For the former, a near‐perfect cross‐validated *R^2 ^* = 0.97 was observed, while for the latter, a more moderate 27% of variance could be explained by the metabolites (Figure 4a,b and Tables S5 and S6). Several sugar signals detected by LC‐MS have been identified as key predictors for either or both of the two laboratory values. Sugars contributing exclusively to the prediction model for glucose level were mainly monosaccharides and related compounds such as the monosaccharide C_6_H_12_O_6_, gulonolactone, and saccharate. Sugars contributing exclusively to the prediction model for HbA1c contained the monosaccharides gluconate and aminohexose, but also the signals obtained for disaccharides and the trisaccharide raffinose. Furthermore, deoxyhexose was identified as a key contributor to the prediction model of HbA1c. Strongest contribution to both models was observed for the small organic acid hydroxymethylglutarate (HMG). Furthermore, both models individually are characterized by contribution of various small organic acids in addition to HMG, such as oxalomalate (contributing to glucose), trans‐aconitate, and citrate (contributing to HbA1c).

Further organ systems being well‐explained by the metabolomics data in association with their clinical parameters include the liver and adipose tissue. Common clinical values representing liver physiology were predicted with an average *R^2 ^* = 0.29 with models for fatty liver index (FLI) being primarily driven by various amino acids such as glutamate, acetyl‐serine, glutamine, asparagine, alanine, betaine, glycine, methylhistidine, and leucine (Table S7). Additional compounds with high contribution to the model included raffinose, kynurenine, and uric acid. Models for fibrosis‐4 (Fib4) score were predominantly contributed to by typical liver‐related compounds such as the bile acids glycocholate and glycochenodeoxycholate, and biliverdin (Table S8). Elastic net regressions developed on the metabolomics data to describe (1) adipose tissue, clinically represented by body mass index and waist‐to‐height‐ratio, and (2) the vascular system, clinically represented by aortic pulse wave velocity, carotid‐femoral pulse wave velocity, aortic augmentation index, ankle‐brachial index, and carotid intima‐media thickness, were able to describe an average of 16.6% of the variance and 15.2% of the variance, respectively.

Next to the description of associations to common, organ‐related clinical traits, we investigated whether the models were suitable to discriminate individuals with diseases of specific organs (Figure 3b). Using metabolomics data as predictors, several disease systems reached a better than random prediction. This was particularly pronounced for the two liver diseases (1) fatty liver disease (FLD) and (2) liver fibrosis, where metabolic models could correctly predict 8 out of 10 patients (composite score of: FLD, 5‐fold cross‐validated AUC: 0.90; liver fibrosis, 5‐fold CV AUC: 0.72) (Figure 4c,d and Tables S9 and S10). Key predictors of FLD include various amino acids such as glutamate, acetyl‐serine, and glutamine. Key predictors of liver fibrosis are, next to others, cystine, an oxidized derivative of cysteine, and the nucleotides adenosine monophosphate and inosine monophosphate. Furthermore, 7 out of 10 patients were correctly predicted for arterial hypertension (AUC = 0.70) and obesity (AUC = 0.73), and 6 out of 10 patients for chronic kidney disease (AUC = 0.61) (Tables S11–S13).

### 2.5 Empagliflozin Reduces Plasma Urate and Deoxyhexose Levels Independent of Fasting Blood Glucose

**FIGURE 5:**
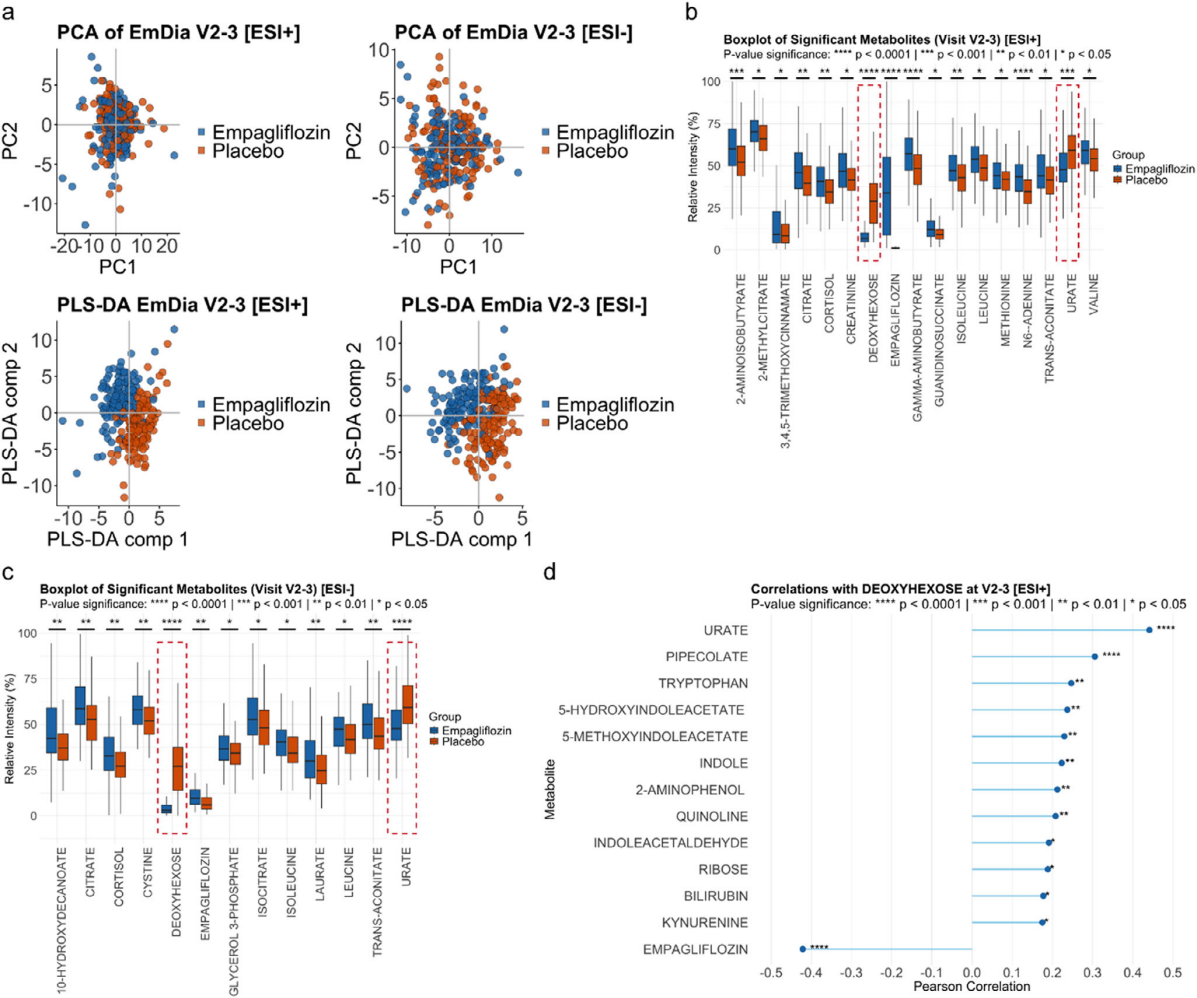
Metabolic differences between empagliflozin and placebo at V2‐3. (a) PCA and PLS‐DA of all samples at V2‐3 acquired in positive ion mode and negative ion mode, color‐coded for placebo and empagliflozin. (b) Boxplots showing metabolites with significant (FDR‐adjusted *p* < 0.05) difference between placebo and empagliflozin using combined data acquired in positive ion modes at V2‐3. Relative intensities are displayed as percentages (Intensity/max (Intensity) × 100) and represent the metabolite signal of a sample scaled to the maximum intensity of that metabolite across all samples for better comparability. Red dashed lines highlight the two metabolites deoxyhexose and urate, further elaborated on in the discussion. (c) Boxplots showing metabolites with significant (FDR‐adjusted *p* < 0.05) difference between the placebo and empagliflozin groups using combined data of V2‐3 as observed in negative ion mode. Relative intensities are displayed as percentages (Intensity/max (Intensity) × 100) and represent the metabolite signal of a sample scaled to the maximum intensity of that metabolite across all samples for better comparability. Red dashed lines highlight the two metabolites deoxyhexose and urate, further elaborated on in the discussion. (d) Correlation plot showing metabolites significantly (*p* < 0.05) correlating with deoxyhexose levels in the dataset acquired for V2‐3 in positive ion mode.

We next focused on identifying biologically relevant changes of the metabolic phenotype across empagliflozin treatment and placebo group using all data obtained for V2 and V3 combined. While principal component analysis (PCA) did not show separation of the empagliflozin and placebo group, partial least squares‐discriminant analysis (PLS‐DA) demonstrated a pronounced separation of the empagliflozin and placebo group in both positive and negative ion mode (Figure 5a). As expected, we found no significantly altered metabolites between the placebo and empagliflozin group at V1, prior to first empagliflozin administration. In contrast, we found significant (FDR‐adjusted *p* values < 0.05) differences in the abundance of 17 metabolites measured in positive ion mode and 13 metabolites measured in negative ion mode between the empagliflozin and the placebo group at V2‐3 (Figure 5b,c). Most strikingly, we found significantly lower levels of urate and a deoxyhexose with the molecular formula of C_6_H_12_O_5_ in the plasma of patients treated with empagliflozin compared to patients receiving a placebo. These effects were consistently observed when acquiring data in positive ion mode and in negative ion mode, validating the reproducibility of our LC‐MS methodology and the significance of the observed metabolic changes. In line with the differential abundance analysis, correlation analysis of deoxyhexose revealed the highest positive correlation with urate and the highest negative correlation with empagliflozin (Figure 5d).

## 3 Discussion

We here describe the development of a scalable LC‐MS‐based metabolomics method allowing for the metabolic characterization of large plasma cohorts such as EmDia. Our methodology is characterized by a reliable metabolite annotation based on spectral libraries obtained from reference compounds of the MSMLS, which has been chosen due to inherent uncertainties in the *de novo* annotation of metabolites based on online spectral libraries like MONA, GNPS, and NIST 2017 [12, 15, 16, 17]. These uncertainties are common when using online repositories, bearing the risk of insufficient spectral similarity due to different instruments or instrument parameters used, and unreliable RT information due to varying chromatographic setups. Within the framework of our methodology, these uncertainties can be largely omitted. Relative metabolite abundances used for clinical analysis are relying on compound annotations backed‐up by synthetic reference standard measurements; however, they do not reflect a fully targeted and therefore a priori limited approach, as spectral libraries obtained from in‐house reference materials can be dynamically expanded and re‐mined at any time.

When assessing the targeted data analysis strategies applied to DDA and DIA results based on MS1, we observed slightly higher results in DDA and DIA analysis. This is surprising, as in both measurement types, the MS1 parameters were kept constant. This is likely attributable to the higher computational load when processing DIA data and the automated peak integration of low‐intensity ion adducts in SCIEX OS, rather than reflecting real differences in the MS1 spectra. This led us to the conclusion to use Skyline instead of SCIEX OS. We were not surprised to see that in an untargeted analysis, where metabolite annotation is exclusively based on MS/MS spectral similarity, DDA outperformed DIA in annotation reproducibility. This is likely because DDA is prone to provide cleaner fragmentation spectra, and spectral matching in DIA still suffers from inherent problems with low fragment ion intensity and deconvolution. It was surprising, however, to see that untargeted analysis of both DDA and DIA did not reach the metabolic depth we were able to achieve through analysis with a well‐curated targeted data analysis in combination with MS1‐based metabolite annotations. This shows that there are still major bottlenecks in the fully untargeted data analysis based on publicly available spectral libraries, which further corroborates our approach of fully relying on metabolite annotations based on reference compounds. For the analysis of cohorts, this observation has been crucial in informing our decision to perform the data analysis of large cohort samples in a targeted way based on MSMLS compounds rather than in an untargeted way, which was implemented in Skyline, known for its good computational performance in cohorts, allowing manual synchronized feature integration for the highest reproducibility [18].

In light of this annotation strategy fully relying on curated spectral libraries based on synthetic standards, in our opinion, the per‐sample annotation via MS/MS matching becomes less crucial. It becomes rather more interesting to further explore potential additional annotations as hypothetical metabolites that can subsequently be validated with synthetic standards. Hence, we also took interest in annotations showing up inconsistently across replicates, prompting us to perform the analysis of distinctive annotations. We were surprised to see that DIA resulted in roughly 31% more annotations than we observed in DDA. While it is not completely clear, whether this is due to an increase in true positives or false positives, it is not inconceivable for DIA to have the edge here, as DIA is aimed to provide an unbiased, more comprehensive fragment ion coverage, potentially resulting in more low‐intensity annotations that are not captured by the stochastic peak picking approach used in DDA. This observation has informed our decision to acquire cohort samples in DIA. While the MS1‐based metabolite annotation of our in‐house MSMLS spectral library by targeted analysis serves as a starting point, the DIA‐LC‐MS analysis of pooled plasma QCs, SRM 1950, and potentially even individual samples will allow for post‐acquisition data mining. Thus, DIA has the potential to generate a larger number of additional candidate annotations than DDA. Over time, these analyses will be used to expand our in‐house spectral library by adding additional compounds to our targeted extraction list, based on newly acquired reference data from synthetic standards. This will allow for targeted re‐evaluation of cohorts with a larger metabolite spectral library in the future, and potentially opening doors for MS2‐based quantification, which would not be possible on DDA data.

When benchmarking our methodology on the EmDia trial using elastic net regression, we found high predictive values for several organ physiologies and pathophysiologies. Metabolites selected by the models based on high predictive power for specific clinical systems, included previously described and well‐established biomarkers, recently suggested biomarker candidates, and novel findings. eGFR values, for instance, were primarily described by metabolic signals known to be markers of kidney function, such as creatinine and kynurenate—a degradation product of tryptophan—and N‐acetylneuraminic acid—known to be filtered but not reabsorbed by the human kidney in a similar fashion to creatinine [19, 20]. Similarly, glucose values were mainly predicted by the signal for the monosaccharide C_6_H_12_O_6_ (which includes glucose, but is analytically not distinguishing other hexoses such as galactose and fructose), and other sugars. Equally, the prediction model for HbA1c values selected various sugar signals. Fibrosis was described by typical liver‐related compounds such as glycocholate, glycochenodeoxycholate, and biliverdin. In correspondence with recent literature suggesting novel biomarker candidates, we observed a high contribution of various amino acids to describe FLI, which aligns with the emergence of using the glutamate‐serine‐glycine (GSG) index as a non‐invasive diagnostic marker of non‐alcoholic fatty liver disease (NAFLD) [21, 22]. As a novel finding, we observed a strong contribution of HMG to the models describing blood glucose and HbA1c. HMG is a precursor of the synthesis of coenzyme Q10, which—to the best of our knowledge—has not been described in this context yet.

When comparing empagliflozin intake‐associated alterations in metabolite abundance at V2‐3 we observed blood glucose levels not to be regulated in an empagliflozin‐dependent manner. This is in line with the inclusion criteria of the EmDia study regarding T2DM with stable glucose‐lowering therapy [5]. The strong group‐specific effect observed for deoxyhexose levels showcases a limitation of our approach, in that it is not capable of differentiating various isobaric sugars. Candidates for the annotated carbohydrate signal are different deoxyhexoses like fucose, which is usually of low plasma concentration, or the exogenous agent 2‐deoxy‐D‐glucose. Both have been demonstrated not to be transported by SGLT2 [23, 24, 25]. Biologically, the most likely source of the signal stems from naturally occurring 1,5‐anhydroglucitol (1,5‐AG), an inert, non‐metabolizable 1‐deoxy polyol of D‐glucose. 1,5‐AG is a validated marker for the prediction of short‐term glycemic changes complementary to fructosamine and HbA1c [26]. This is in line with our observation of deoxyhexose as a key predictor of HbA1c at V1 prior to SGLT2 inhibition. 1,5‐AG enters the kidney via the glomerulus and is reabsorbed via renal proximal tubules, where it competes with glucose for transport into the bloodstream, presumably via SGLT4 and SGLT5 [27]. Under hyperglycemic conditions with glucosuria, the steady state of 1,5‐AG is disrupted and high blood glucose levels competitively inhibit the tubular reabsorption, leading to lowered 1,5‐AG levels in the blood until the end of the hyperglycemic state [28]. Similarly, glucosuria induced by SGLT2 inhibition reduces tubular absorption of 1,5‐AG, independent of systemic hyperglycemia [29]. The reduced 1,5‐AG plasma levels in patients with T2DM with stable glucose‐lowering background therapy under additional administration of empagliflozin is in accordance with findings of a recent study by Kappel et al., suggesting an inhibited reabsorption of 1,5‐AG due to SGLT2 inhibition, independent of blood glucose levels [30].

Our findings of significantly lowered urate plasma levels in the empagliflozin group at V2‐3 support the findings of the EMPA‐REG and the EmDia study [6, 7]. This is likely a secondary effect of the increased glucose level in the tubular fluid due to SGLT2‐inhibition‐induced glucosuria and an unknown interplay between GLUT9 or the urate transporter URAT1 [31]. This could be verified in future dedicated follow‐up experiments analyzing urine samples of the EmDia cohort or similar cohorts investigating the effects of SGLT2 inhibition. Lowered urate levels have been associated with improved CVD outcomes in patients with T2DM in EMPA‐REG sub‐analyses [32, 33]. Altogether, these findings may partially explain the observed improvements in CVD outcomes; however, further investigation is required to establish a definitive cause‐and‐effect relationship.

## 4 Conclusions

In conclusion, the metabolic phenotyping of large clinical cohorts is of ever‐increasing interest in the research field of biomarker discovery. To date, fully untargeted approaches for confident metabolite annotation per sample remain challenging for large cohort studies due to limitations of processing power. Moreover, fully untargeted annotation based on public spectral libraries bears the risk of low‐confidence and erroneous annotations, while fully targeted approaches remain blind to the discovery of compounds not a priori included in the analysis. We here present a pragmatic approach how to deal with these obstacles. Our methodology relies on an easy‐to‐use sample preparation protocol in combination with 12 min LC‐MS analysis time per sample and ion mode. Metabolite annotation is driven by the use of an in‐house spectral library used for targeted data extraction, that is dynamically expanded based on project‐specific compounds of interest and regularly occurring additional untargeted annotations. Used for the analysis of plasma samples of the EmDia cohort, we benchmarked the predictive power of the metabolomics methodology based on a large set of clinical data available for the same patients. By employing elastic net regression, we could show the highest predictive power of the metabolomics data specifically for physiological traits that are clinically already defined by small molecule measurements, most prominently by achieving a near perfect cross‐validated *R^2 ^* = 97% for clinical glucose measurements. Moreover, several known or recently proposed disease‐specific or organ function‐specific biomarkers were strong predictors in our data as well, such as creatinine, kynurenic acid, and uric acid for the prediction of kidney function, and several bile acids combined with biliverdin for the prediction of liver fibrosis. We could corroborate the recently suggested GSG‐index as a marker for FLI. When applied to differentiate between empagliflozin‐ and placebo‐treated individuals in the context of EmDia, metabolomics analysis revealed distinct alterations in the abundance of several metabolites. We found significantly reduced 1,5‐AG (deoxyhexose) levels under SGLT2 inhibition, despite unchanged glycemic status. Furthermore, our data support the potential association between reduced plasma urate levels and improved cardiovascular outcomes. Altogether, the metabolomics workflow presented here provides a fast and clinically reliable framework for high‐throughput metabolic phenotyping of plasma cohorts, characterized by scalability to cohorts of thousands to ten thousand of samples, due to its short per‐sample analysis time and the computationally low‐demanding data analysis strategy.

## 5 Methods

### 5.1 Chemicals and Reagents

All solvents used for this study were of high‐quality LC‐MS grade. LC‐MS grade water (H_2_O), acetonitrile (ACN), and methanol (MeOH) were bought from AppliChem GmbH (Darmstadt, Germany) and formic acid (FA) and isopropanol from Merck KGaA (Darmstadt, Germany). We acquired the following chemicals and plasma standards MSMLS‐LOT‐230‐16, SRM 1950, 2‐deoxy‐d‐glucose, metformin‐hydrochlorid, N‐Methyl‐2‐pyridone‐5‐carboxamide, N‐Methyl‐4‐pyridone‐5‐carboxamide and ammonium acetate from Merck KGaA (Darmstadt, Germany) and empagliflozin from Adipogen AG (Fuellinsdorf, Switzerland). The Kinetex 2.6 µm F5 100 Å, LC Column 150 × 2.1 mm was bought from Phenomenex (Aschaffenburg, Germany). Eppendorf Safe‐lock tubes 1.5 mL, Eppendorf Deepwell Plate 96/1000 µL, Eppendorf Storage Foil and Eppendorf Microplate 96/V‐PP were bought from Eppendorf (Hamburg, Germany). Sapphire pipette tips were bought from Greiner Bio‐One GmbH (Frickenhausen, Germany).

### 5.2 EmDia Study

EmDia is a single‐center, randomized, double‐blind, two‐arm, placebo‐controlled, parallel group study of phase IV (ClinicalTrials.gov; NCT02932436. Registration date, 13/10/2016). All study documents were approved by the local ethics committee and data protection officer prior to study initiation. All participants provided written informed consent, and the study was conducted in accordance with the Declaration of Helsinki and recommendations for Good Clinical Practice.

### 5.3 Samples

SRM 1950 was used for the development of the metabolomics workflow of this study. Blood samples of the *N* = 144 EmDia (ClinicalTrials.gov, unique identifier: NCT02932436) study participants were collected in EDTA blood collection tubes at three different time points as part of their clinical examination at the University Medical Center of the Johannes Gutenberg University Mainz, including the first visit before randomization, one week after and after 12‐weeks. The collected blood was centrifuged for 10 min at 1780 × *g* for isolation of plasma, immediately aliquoted and transferred to −80°C within a maximum of 2 h after blood draw, during which the samples were kept at room temperature. Aliquots of the randomized and anonymized plasma samples were transferred from the biobank to the laboratory on dry ice and then stored at −80°C at all times in the laboratory until sample preparation.

### 5.4 Metabolite Extraction and QCs

Metabolites were extracted from plasma using single‐phase MeOH‐based protein‐precipitation combined with centrifugation. Typically, 88 cohort samples per batch were thawed at 4°C in a 96‐well bio‐bank vial format, and 25 µL of plasma were transferred to a 96‐well SBS deep‐well plate and diluted in 45 µL LC‐MS grade H_2_O at 4°C. 210 µL of −20°C cold MeOH were added, followed by shaking at 1000 rpm at 4°C for 5 min. Samples were then incubated at −20°C for 2 h and subsequently centrifuged at 4800 × g at 4°C for 30 min. 150 µL of the supernatant was transferred into a new 96‐well plate and dried under constant nitrogen flow. Precipitates were reconstituted in 100 µL H_2_O and subsequently shaken for 5 min at 300 rpm at 4°C. Samples were then split into three aliquots, and a batch‐wide plasma QC was pooled. Sample preparation of SRM 1950 plasma followed the same protocol, yet was performed in 1.5 mL Eppendorf tubes instead.

### 5.5 LC‐MS/MS

LC‐MS analysis was conducted on an Agilent 1290 Infinity II (Agilent, Waldbronn, Germany) coupled to a SCIEX ZenoTOF 7600 equipped with the SCIEX Optiflow ESI source (SCIEX, Darmstadt, Germany). RP chromatographic separation of metabolites was performed using the Phenomenex Kinetex F5 2.6 µm (2.1 mm × 150 mm). Mobile phase A consisted of H_2_O + 0.1% FA, and mobile B consisted of ACN + 0.1% FA. Metabolite samples were loaded onto the column for analysis in positive ion mode and negative ion mode, respectively, using mobile phase A. After trapping, the elution using our 12 min LC‐method started with 0% B at 200 µL/min until 2.1 min, increased to 95% B until 6.5 min, followed by 95% B until 8 min. At 8 min, flow was switched to offline mode via an external post‐column switch valve, and the flowrate was increased to 1 mL/min until 8.25 min, was maintained until 9.25 min, and then changed to 0 % B until 9.50 min. Flowrate was decreased to 200 µL/min until 10 min and kept until 12 min for pressure stabilization and re‐equilibration of the column. The column compartment was maintained at 50°C throughout the batch and the multisampler temperature was kept at 4°C.

Ion source & gas parameters were the same for the DDA and DIA methods with ion source gas 1/2: 45 psi; curtain gas: 35 psi; CAD gas 7; and temperature: 400°C. The spray voltage was set to 5000 V (−4500 V), with a 100 ms TOF MS accumulation time, a mass range set to 70–1000 *m/z*, a declustering potential of (−)80 V, and a collision energy of (−)10 V, respectively for positive and negative ion mode. TOF MS/MS mass range was set to 40–1000 *m/z* with activated Zeno pulsing with a threshold of 20,000 cps for both DDA and DIA. Accumulation time for each of the 30 mass windows for the DIA method was 0.005 s, with a collision energy for CID of (−)20 V for molecules up to 250 Da and (−)35 V for molecules larger than 250 Da, respectively. Optimal Q1 isolation width for the 30 DIA mass windows was determined by the SCIEX SWATH window calculator in Microsoft Excel based on a peak intensity list obtained from a TOF MS measurement of a plasma extract. Accumulation time of the TOP 11 DDA MS/MS experiments was set to 0.025 s, with a DP of ±50 V and a collision energy for CID of 20 ± 15 V or 35 ± 15 V for positive and −20 ± −15 V or −35 ± −15 V for the negative ion mode.

### 5.6 MSMLS Spectral Library Generation

For the initial generation of a spectral library, we used the MSMLS, containing 612 small molecule standards. MSMLS compounds were prepared and injected according to manufacturer recommendations. In brief, polar compounds were dissolved by adding 7.5 µL of LC‐MS grade MeOH and 142.5 µL of LC‐MS grade H_2_O. Carbohydrates were dissolved in 250 µL of pure H_2_O. Lipid‐like structures were dissolved in 150 µL of a 1:1 MeOH:chloroform (CHCl_3_) solvent mixture. Except for carbohydrates, compounds were pooled row‐wise for spectral library creation. All standards were measured in both ion modes, using the DDA settings described above. For measurement in positive ion mode and for measurement in negative ion mode, 4 and 6 µL were injected, respectively. Using a targeted feature annotation in SCIEX analytics based on the compounds’ molecular formulas and precursor *m/z* as [M+H]+ precursor adduct, we generated a MSMLS spectral library in SCIEX library viewer based on high purity DDA MS2 data. We applied the same approach and methodology for additional reference standards from untargeted metabolomics results.

### 5.7 Comparing DIA vs. DDA Targeted and Untargeted Approaches

Five injection replicates of SRM 1950 were measured in both DDA and DIA for comparison and evaluation of targeted and untargeted data analysis. An untargeted spectral library search against our in‐house spectral library and the commercial NIST 2017 spectral library was performed separately for DDA and DIA data in SCIEX Analytics, using the MQ4 peak integration algorithm, with an area‐ratio‐threshold of unknown‐to‐control of 3, a precursor mass tolerance of 0.04 Da, and a library hit annotation acceptance score of ≥ 50. Targeted evaluation was performed using molecular formula, precursor *m/z*, and explicit RTs of the metabolites, based on our in‐house spectral library, allowing mass errors of up to 10 ppm and RT shifts of up to 20%. We defined “replicate annotation” as annotations being present across all five samples and “distinctive annotations” as being annotated in a minimum of two samples. We omitted annotations being present in just one sample to reduce the number of miss‐annotations.

### 5.8 EmDia Cohort Batch Design

We implemented a batch design including several QCs for batch correction and untargeted metabolite annotation for in‐house compound library extension. Injection volume used for all measurements were 1 µL in positive ion mode and 2 µL in negative ion mode. Following blank injections, conditioning of the column was achieved by injecting an extract of a plasma sample obtained from the local blood bank. At the beginning and end of each batch, pooled QCs were injected as well as every eight samples. Every batch was ended with the injection of blanks. Separate processing blanks were regularly acquired to ensure not to annotate background ions. We employed in‐batch calibration via the X500 calibration solutions for the ZenoTOF every 10^th^ sample to achieve continuous high instrument accuracy throughout the measurements. Using our 12 min LC‐MS analysis methodology, this enabled a throughput of up to 120 injections per day, with a batch consisting of typically 88 samples plus QCs, conditioning, and blanks. The separate measurement of the EmDia cohort, first in positive ion mode, followed by negative ion mode, represented a total of ∼1400 injections, which were measured over the course of less than 2 weeks.

### 5.9 In‐House Library Expansion

To expand our in‐house spectral library with molecules not present in the MSMLS metabolite library, we initially performed untargeted spectral library searches against the commercial NIST 2017 spectral library on pooled plasma QC samples and SRM 1950 in both positive and negative ion mode separately. We initially focused on purchasing standard compounds for metabolite annotations that were of potential interest with respect to the study. Standard compounds for metformin, empagliflozin, glycerophosphocholine, N‐methylarginine, and N‐methyl‐2‐pyridone‐5‐carboxamide were procured, and used for spectral library expansion, using analogous methodology as for MSMLS described above.

### 5.10 Feature Extraction and Annotation

For targeted feature extraction and annotation of metabolites in both ion modes separately, we created two transition lists including molecular formula and the RT, as recorded in our in‐house spectral library. These lists contained MSMLS reference standards merged with authentic reference standards added during MS/MS library expansion. The EmDia plasma cohort data files were then processed in Skyline (version 22.2.0.527), based on generating XICs using the orthogonal information of precursor *m/z* mass accuracy and explicit RT information included in the transition list [34]. Adducts included were [M+H]+, [M+K]+, and [M+Na]+ in positive and [M‐H]− in negative ion mode. We used high‐selectivity extraction and included up to three isotope counts, with parameters for the precursor mass analyzer set to TOF with a resolving power of 30,000. Since metabolite RTs were stable across cohort measurement of each ion mode (Figure S9), we performed manual synchronized peak integration based on RTs observed for the compounds in the pooled plasma QCs across the whole cohort of each ion mode, respectively. Area under the curve values were exported and subjected to peak area normalization and batch correction using the Quality Control‐Robust Spline Correction (QC‐RSC) algorithm with CV assessment pre‐ and post‐batch‐correction (Figures S2 and S10) [35].

### 5.11 Statistical Analysis

Elastic net‐regularized linear and logistic regression models, with respectively 10‐fold and 5‐fold cross‐validation, were fit to identify metabolites associated with continuous traits representing organ function/structure, as well as metabolites capable of discriminating individuals with vs. without disease affecting specific organ systems. For each model, the (cross‐validated) variance explained in the trait by the metabolites (*R^2^*), or the proportion of cases correctly identified as such among all possible case‐control pairs (area under the receiver operating characteristic curve, AUC), were recorded. Normalized data of V2 and V3 was used for multivariate and univariate statistical data analysis to identify metabolic changes in regard to the empagliflozin treatment in the statistical software R (version 4.2.3) with correction for multiple testing using FDR‐adjusted *p* < 0.05.

## Author Contributions

Conceptualization: Fabian Schmitt and Thierry Schmidlin. Methodology: Fabian Schmitt, Vincent ten Cate, Zlatka Fischer, and Mathias Hagen. Data analysis: Fabian Schmitt, Vincent ten Cate, and Zlatka Fischer. Investigation: Fabian Schmitt, Vincent ten Cate, Zlatka Fischer, Mathias Hagen, and Barbara A. Steigenberger. Resources: Philipp S. Wild and Thierry Schmidlin; Writing—original draft: Fabian Schmitt. Writing—review & editing: Fabian Schmitt, Vincent ten Cate, and Thierry Schmidlin. Visualization: Fabian Schmitt and Vincent ten Cate. Supervision: Stefan Tenzer, Philipp S. Wild, and Thierry Schmidlin. Project administration: Thierry Schmidlin. Funding acquisition: Stefan Tenzer, Philipp S. Wild, and Thierry Schmidlin.

## Funding

Thierry Schmidlin is funded by the German Federal Ministry of Research, Technology and Space as part of the DIASyM project under grant number 031L0218. Philipp S. Wild is funded as part of the DIASyM project under grant number 161L0217A. The EmDia trial is an academic, GCP sponsor‐investigator clinical trial funded with support of Boehringer Ingelheim. Philipp S. Wild receives additional funds by the Federal Ministry of Research, Technology and Space 01EO1503.

## Ethics Statement

No unexpected or unusually high safety hazards were encountered. EmDia is a single‐center, randomized, double‐blind, two‐arm, placebo‐controlled, parallel group study of phase IV (ClinicalTrials.gov; NCT02932436. Registration date, 13/10/2016). All study documents were approved by the local ethics committee and data protection officer prior to study initiation. All participants provided written informed consent, and the study was conducted in accordance with the Declaration of Helsinki and recommendations for Good Clinical Practice.

## Conflicts of Interest

The authors declare no conflicts of interest.

## Supporting information




**Supporting File**: pmic70075‐sup‐0001‐SuppMat.docx.

## Data Availability

The data underlying the study are not publicly available due to data protection regulations of the clinical trial. To meet the general idea of verification and reproducibility of scientific findings, we offer access to data at the local database in accordance with the ethics vote on request via the corresponding author. All wiff2 files of SRM 1950 standards are available on Peptide Atlas via dataset identifier PASS05920. The *R* code used for batch‐normalization, univariate and comparative statistical data analysis is available on GitHub (https://github.com/schmittf‐lcms/EmDia‐R‐Code).
