# Supplementary material for: Metabolic Profiling of the EmDia Cohort by LC‐MS Reveals Empagliflozin‐Intake Associated Regulation of 1,5‐anhydroglucitol and Urate
Source: Proteomics. 2025 Dec 3;26(1):44–56. doi: 10.1002/pmic.70075 (PMC12809005; doi:10.1002/pmic.70075)
Supplement: Supplementary file 1 — Supporting File: pmic70075‐sup‐0001‐SuppMat.docx. [file PMIC-26--s001.docx]

**Supporting Information for**

**Metabolic Profiling of the EmDia Cohort by LC-MS Reveals Empagliflozin-intake Associated Regulation of 1,5-anhydroglucitol and Urate**

Fabian Schmitt^a^, Vincent ten Cate^b,c,d^, Zlatka Fischer^b^, Mathias Hagen^a^, Barbara A. Steigenberger^e^, Stefan Tenzer^a,f^, Philipp S. Wild^b,c,d,g^, Thierry Schmidlin^a,d,f,h^

**Affiliations**

1. Institute for Immunology, University Medical Center, Johannes Gutenberg University Mainz, Mainz, Germany
2. Preventive Cardiology and Preventive Medicine, Dept. of Cardiology, University Medical Center of the Johannes Gutenberg University Mainz, Mainz, Germany
3. Clinical Epidemiology and Systems Medicine, Center for Thrombosis and Hemostasis (CTH), Mainz, Germany
4. German Center for Cardiovascular Research (DZHK), Partner Site Rhine Main, Mainz, Germany
5. Mass Spectrometry Core Facility, Max Planck Institute of Biochemistry, Martinsried, Germany
6. Research Center for Immunotherapy (FZI), University Medical Center of the Johannes-Gutenberg University, Mainz, Germany
7. Institute for Molecular Biology (IMB), Mainz, Germany
8. Correspondence to: schmidlt@uni-mainz.de

**Supplemental Figures**

[Figure S 1. Violin plot of CVs of 79 annotated metabolites from an untargeted spectral library search of plasma extracts of 12 technical replicates that were prepared by methanol (MeOH) based protein-precipitation combined with centrifugation at 4800g for 30min at 4°C. 5](file:///Z:\Publications\Fabian%20-%20EmDia%202024\Wiley%20Proteomics%20Revisions\Trackchanges\Schmitt_et_al_2025_Supporting_edited.docx#_Toc212110600)

[Figure S 2 Boxplots of median CVs of QCs and sample of the EmDia cohort measurement of (a) non-corrected positive ion mode data, (b) non-corrected negative ion mode data, (c) positive ion mode QC-RSC batch-corrected data and (d) negative ion mode QC-RSC batch-corrected data. 6](#_Toc212110601)

[Figure S 3. (a) Chemical subclassification of the MSMLS metabolite standard library used for the development of our targeted metabolomics workflow and (b) of the common metabolites successfully detected in SRM 1950 plasma when performing targeted annotation SCIEX Analytics based on the MSMLS spectral library. 7](#_Toc212110602)

[Figure S 4. Visualization of chromatographic separation obtained for several pairs of isomeric compounds using the Kinetex F5 column: (a) XICs of isoleucine (top) and leucine (middle) obtained for MSMLS standards compared to the XIC of isoleucine and leucine as measured in an SRM 1950 plasma extract (bottom), (b) XICs of betaine (top) and valine (middle) obtained for MSMLS standards compared to the XICs of betaine and valine as measured in an SRM 1950 plasma extract (bottom) and (c) XICs of theobromine (top) and theophylline (middle) obtained for MSMLS standards compared to the XICs of theobromine and theophylline as measured in an SRM 1950 plasma (bottom). 8](#_Toc212110603)

[Figure S 5 Venn-diagram of distinctive (2 out of 5) untargeted annotations of DDA data (blue) and DIA data (orange) across five SRM 1950 technical replicate injections matched by annotation name. 9](#_Toc212110604)

[Figure S 6 Barplot of matched annotations in SCIEX OS of five technical replicate injections of NIST SRM 1950 extracts, with 2 out of 5, 3 out of 5, 4 out of 5 and 5 out of 5 occurrences in either targeted (left) or untargeted (right) feature annotation comparing DDA (blue) and DIA (orange) mode. 9](#_Toc212110605)

[Figure S 7 Barplot of average metabolite annotation counts across five SRM 1950 technical replicate injections using targeted (left) and untargeted (right) processing of DDA (blue) and DIA (orange) data. 10](#_Toc212110606)

[Figure S 8. Bar chart of normalized intensity values of empagliflozin measured in positive ion mode data at V2 (a) and V3 (b), comparing empagliflozin and placebo groups. Error bars represent standard deviation. 11](#_Toc212110607)

[Figure S 9. Chromatographic retention time (RT) stability across batches shown for the selected metabolites creatine, urate, phenylalanine and bilirubin. Integration boundary and peak apex are shown for first and last QC QC injection of the EmDia cohort measurement in positive ion mode (top panels). Analogous data is shown for negative ion mode (bottom). RT deviation remained below 0.1 min over the course of 700 injections on the same Kinetex F5 column in both ion modes. 12](#_Toc212110608)

[Figure S 10. Batch correction of the EmDia cohort measurement of (a) positive ion mode data and (b) negative ion mode data. Principal component analysis of non-corrected raw data of five batches, including samples and QCs (left panels), PCA of batch-corrected data of five batches including samples and QCs (right panels). Batch correction was performed using the Quality Control-Robust Spline Correction (QC-RSC).^1^ 13](#_Toc212110609)

**Supplemental Tables**

[Table S 1. Metabolites used for Skyline transition list for the annotation of metabolites in the EmDia cohort in positive and/or negative ion mode (inclusion indicated in green). ***Added as annotation obtained by untargeted analysis, reference standard not measured yet. 10](#_Toc198033603)

[Table S 2. Log_2_ fold change of annotated metabolites from positive ion mode data comparing placebo and empagliflozin groups using time points V2-V3. Significance is indicated using FDR adjusted *p*-values (**** *p* < 0.0001 | *** *p* < 0.001 | ** *p* < 0.01 | * *p* < 0.05). 12](#_Toc198033604)

[Table S 3 Log_2_ fold change of annotated metabolites from negative ion mode data comparing placebo and empagliflozin groups using time points V2-V3. Significance is indicated using FDR adjusted *p*-values (**** *p* < 0.0001 | *** *p* < 0.001 | ** *p* < 0.01 | * *p* < 0.05). 15](#_Toc198033605)

[Table S 4: Elastic Net regression model (linear relationship): estimated glomerular filtration rate (egfr) ~ Metabolites at BL; 10 fold cv *R^2^* = 0.6233, (simple *R^2^* = 0.8036), N=136, minimal lambda = 2.017 (10 fold-CV), Selected markers 34 17](#_Toc198033606)

[Table S 5: Elastic Net regression model (linear relationship): glucose ~ Metabolites at BL; 10 fold cv *R^2^* = 0.9697, (simple *R^2^* = 0.9841), N=136, minimal lambda = 1.397 (10 fold-CV), Selected markers 32 18](#_Toc198033607)

[Table S 6: Elastic Net regression model (linear relationship): hba1c ~ Metabolites at BL; 10 fold cv *R^2^* = 0.2749, (simple *R^2^* = 0.5019), N=136, minimal lambda = 0.186 (10 fold-CV), Selected markers 20 18](#_Toc198033608)

[Table S 7: Elastic Net regression model (linear relationship): fatty liver index (fli) ~ Metabolites at BL; 10 fold cv *R^2^* = 0.4069, (simple *R^2^* = 0.7460), N=136, minimal lambda = 2.778 (10 fold-CV), Selected markers 43 19](#_Toc198033609)

[Table S 8: Elastic Net regression model (linear relationship): fibrosis-4 score (fib4) ~ Metabolites at BL; 10 fold cv *R^2^* = 0.3170, (simple *R^2^* = 0.7419), N=136, minimal lambda = 0.132 (10 fold-CV), Selected markers 35 20](#_Toc198033610)

[Table S 9: Elastic Net regression model (linear relationship): fatty liver disease (fld) ~ Metabolites at BL; 5 fold cv AUC= 0.9038, (simple AUC = 0.9994), N=142, minimal lambda = 0.027 (5 fold-CV), Selected markers 55 20](#_Toc198033611)

[Table S 10: Elastic Net regression model (linear relationship): liver fibrosis (fib4_bin) ~ Metabolites at BL; 5 fold cv AUC= 0.7249, (simple AUC = 0.9987), N=142, minimal lambda = 0.044 (5 fold-CV), Selected markers 57 21](#_Toc198033612)

[Table S 11: Elastic Net regression model (linear relationship): hypertension ~ Metabolites at BL; 5 fold cv AUC= 0.7011, (simple AUC = 0.9231), N=142, minimal lambda = 0.08 (5 fold-CV), Selected markers 14 22](#_Toc198033613)

[Table S 12: Elastic Net regression model (linear relationship): obesity (adipositas) ~ Metabolites at BL, 5 fold cv AUC= 0.7307, (simple AUC = 0.8591), N=142, minimal lambda = 0.132 (5 fold-CV), Selected markers 18 23](#_Toc198033614)

[Table S 13: Elastic Net regression model (linear relationship): chronic kidney disease (cdk) ~ Metabolites at BL; 5 fold cv AUC= 0.6050, (simple AUC = 0.8418), N=139, minimal lambda = 0.108 (5 fold-CV), Selected markers 10 23](#_Toc198033615)

**
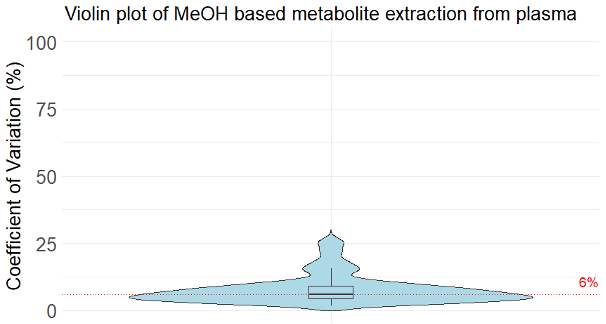
**

Figure S 1. Violin plot of CVs of 79 annotated metabolites from an untargeted spectral library search of plasma extracts of 12 technical replicates that were prepared by methanol (MeOH) based protein-precipitation combined with centrifugation at 4800g for 30min at 4°C.


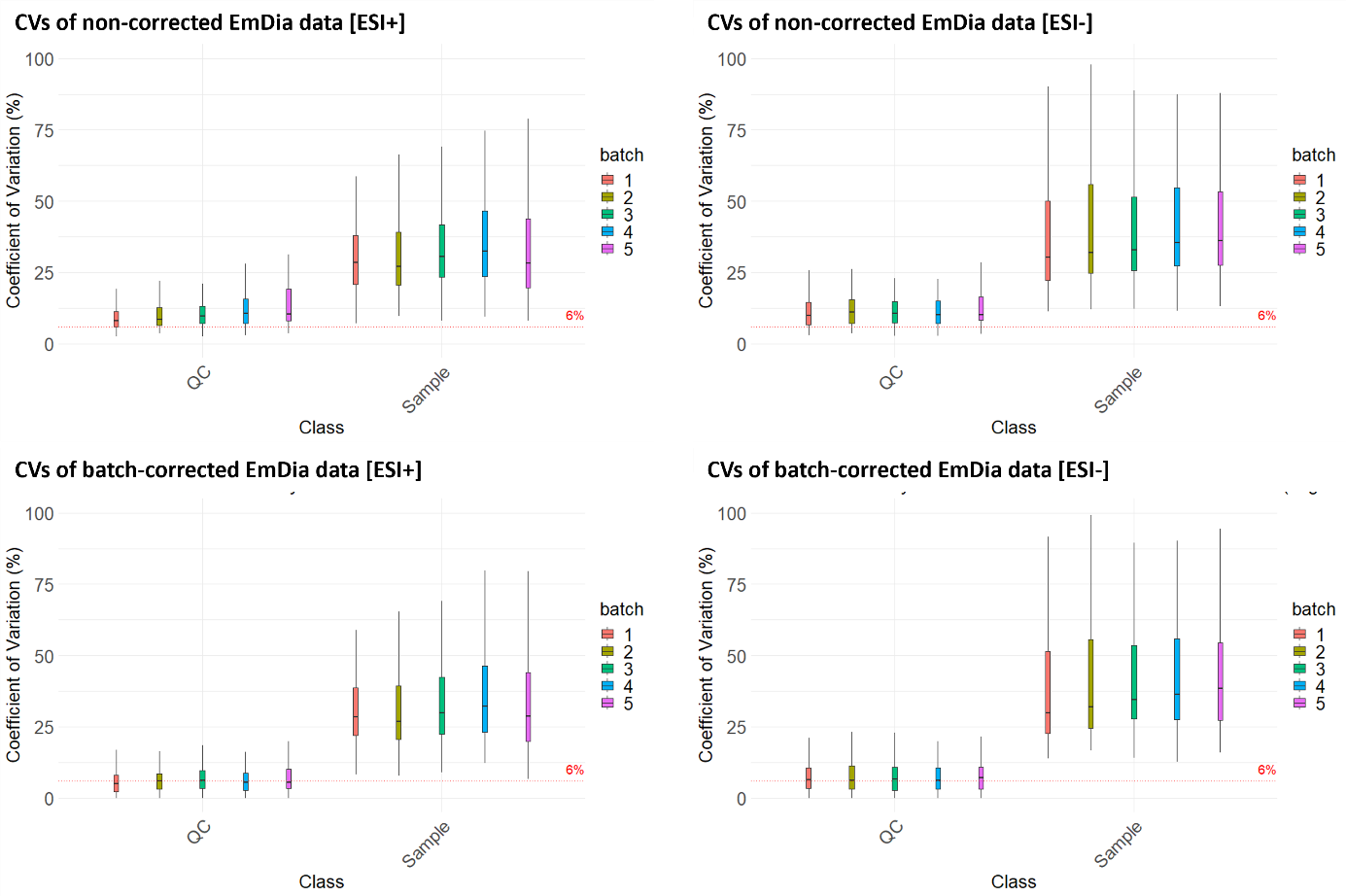


Figure S 2 Boxplots of median CVs of QCs and sample of the EmDia cohort measurement of (a) non-corrected positive ion mode data, (b) non-corrected negative ion mode data, (c) positive ion mode QC-RSC batch-corrected data and (d) negative ion mode QC-RSC batch-corrected data.


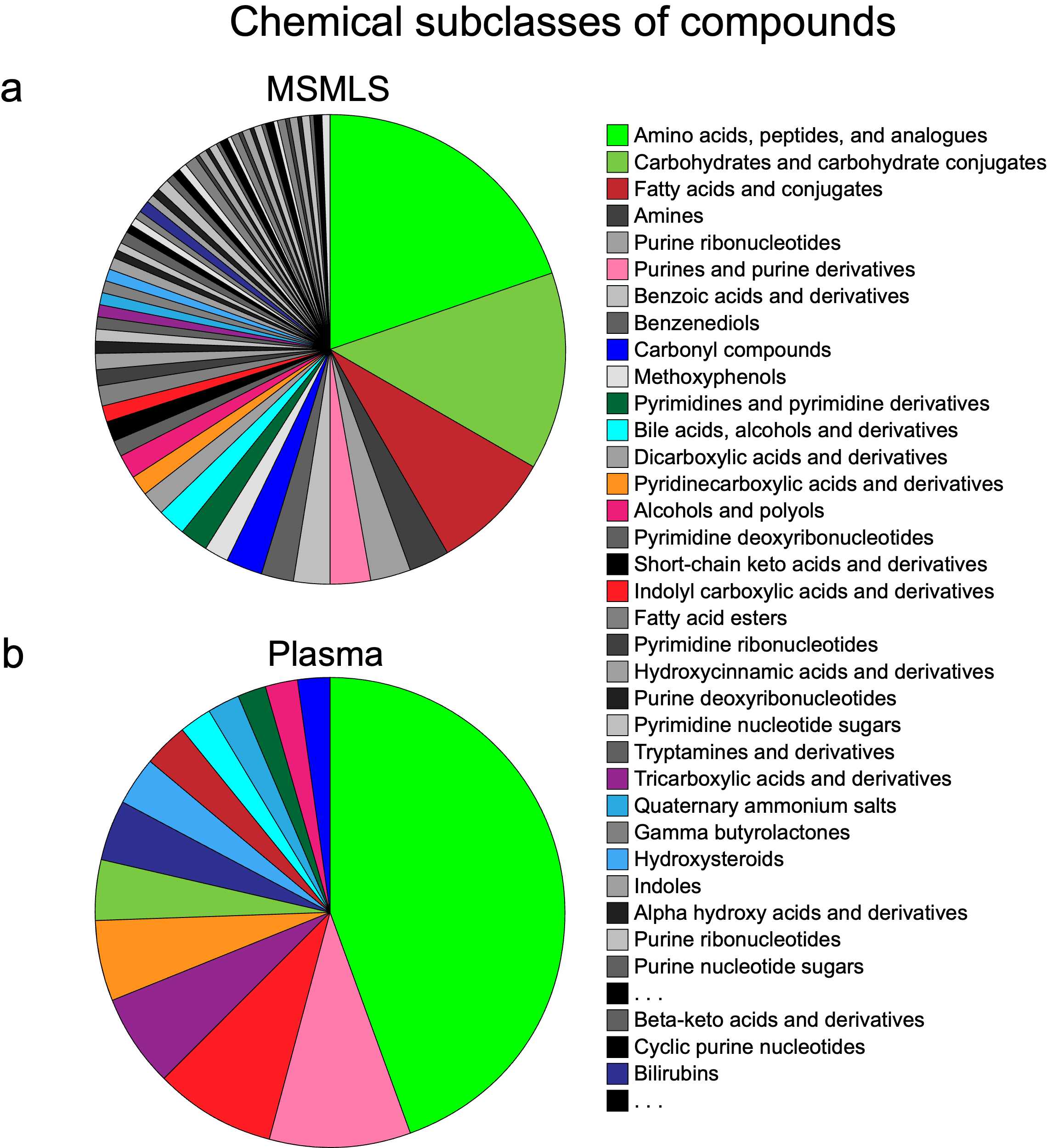


Figure S 3. (a) Chemical subclassification of the MSMLS metabolite standard library used for the development of our targeted metabolomics workflow and (b) of the common metabolites successfully detected in SRM 1950 plasma when performing targeted annotation SCIEX Analytics based on the MSMLS spectral library.

**
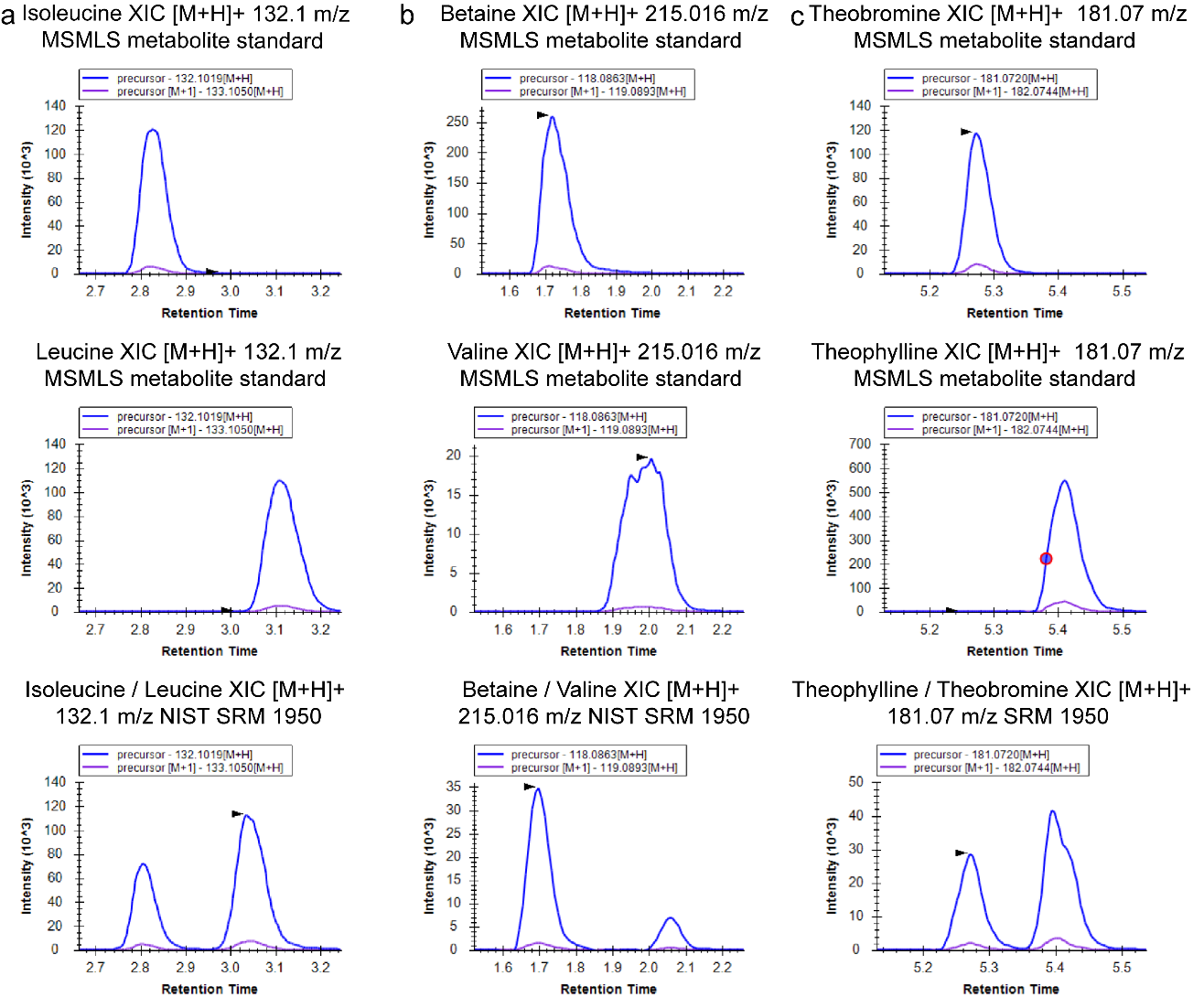
**

Figure S 4. Visualization of chromatographic separation obtained for several pairs of isomeric compounds using the Kinetex F5 column: (a) XICs of isoleucine (top) and leucine (middle) obtained for MSMLS standards compared to the XIC of isoleucine and leucine as measured in an SRM 1950 plasma extract (bottom), (b) XICs of betaine (top) and valine (middle) obtained for MSMLS standards compared to the XICs of betaine and valine as measured in an SRM 1950 plasma extract (bottom) and (c) XICs of theobromine (top) and theophylline (middle) obtained for MSMLS standards compared to the XICs of theobromine and theophylline as measured in an SRM 1950 plasma (bottom).

**
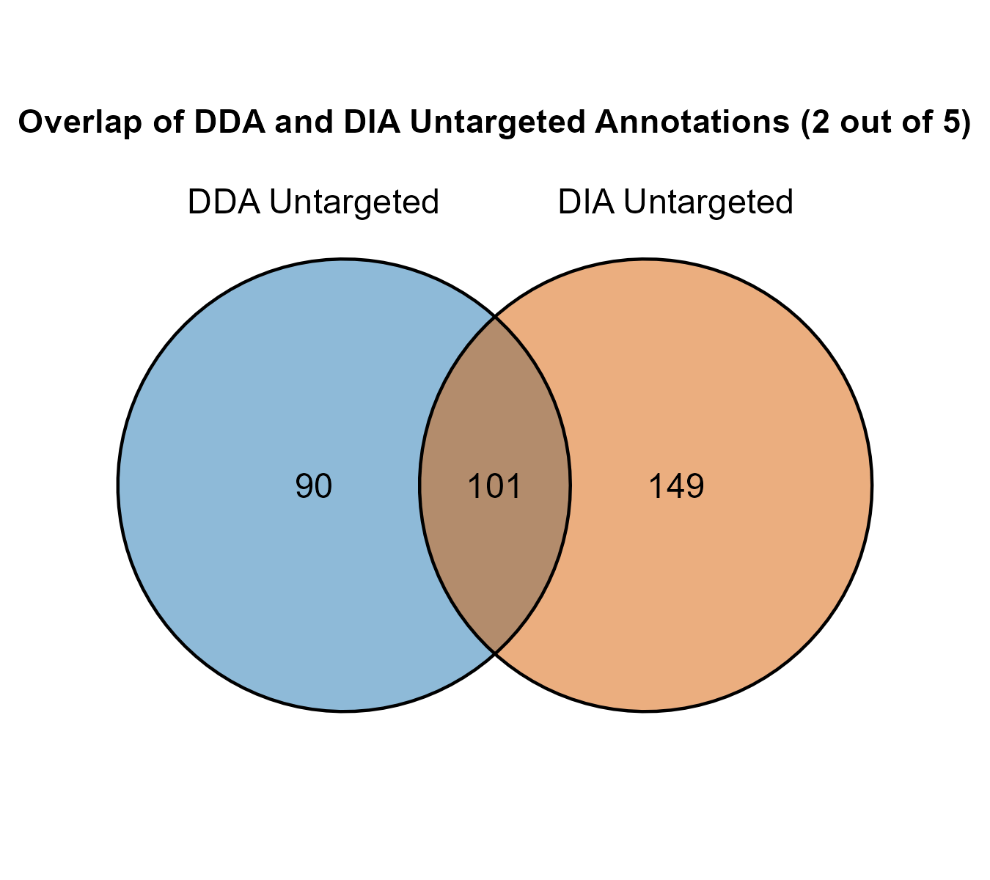
**

Figure S 5 Venn-diagram of distinctive (2 out of 5) untargeted annotations of DDA data (blue) and DIA data (orange) across five SRM 1950 technical replicate injections matched by annotation name.


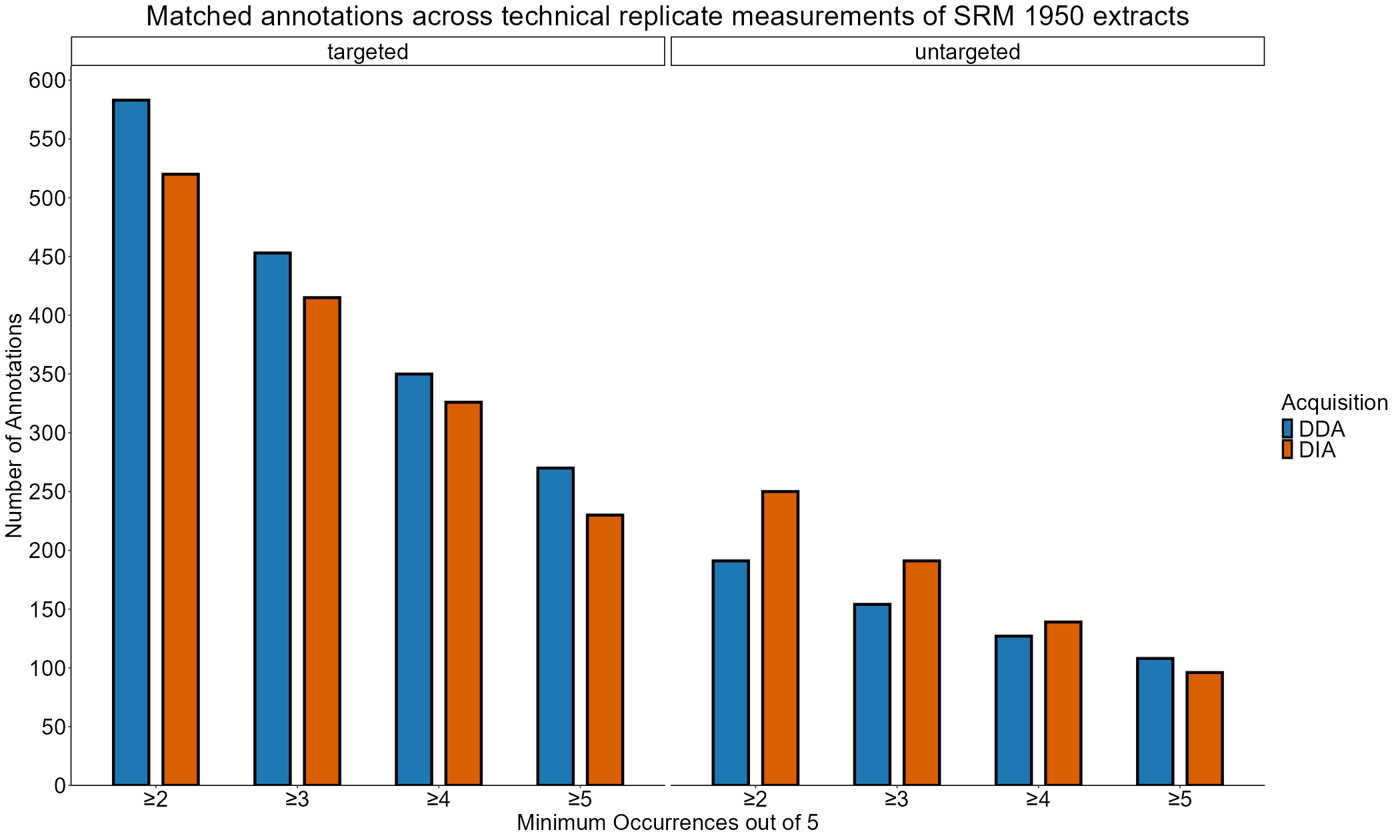


Figure S 6 Barplot of matched annotations in SCIEX OS of five technical replicate injections of NIST SRM 1950 extracts, with 2 out of 5, 3 out of 5, 4 out of 5 and 5 out of 5 occurrences in either targeted (left) or untargeted (right) feature annotation comparing DDA (blue) and DIA (orange) mode.


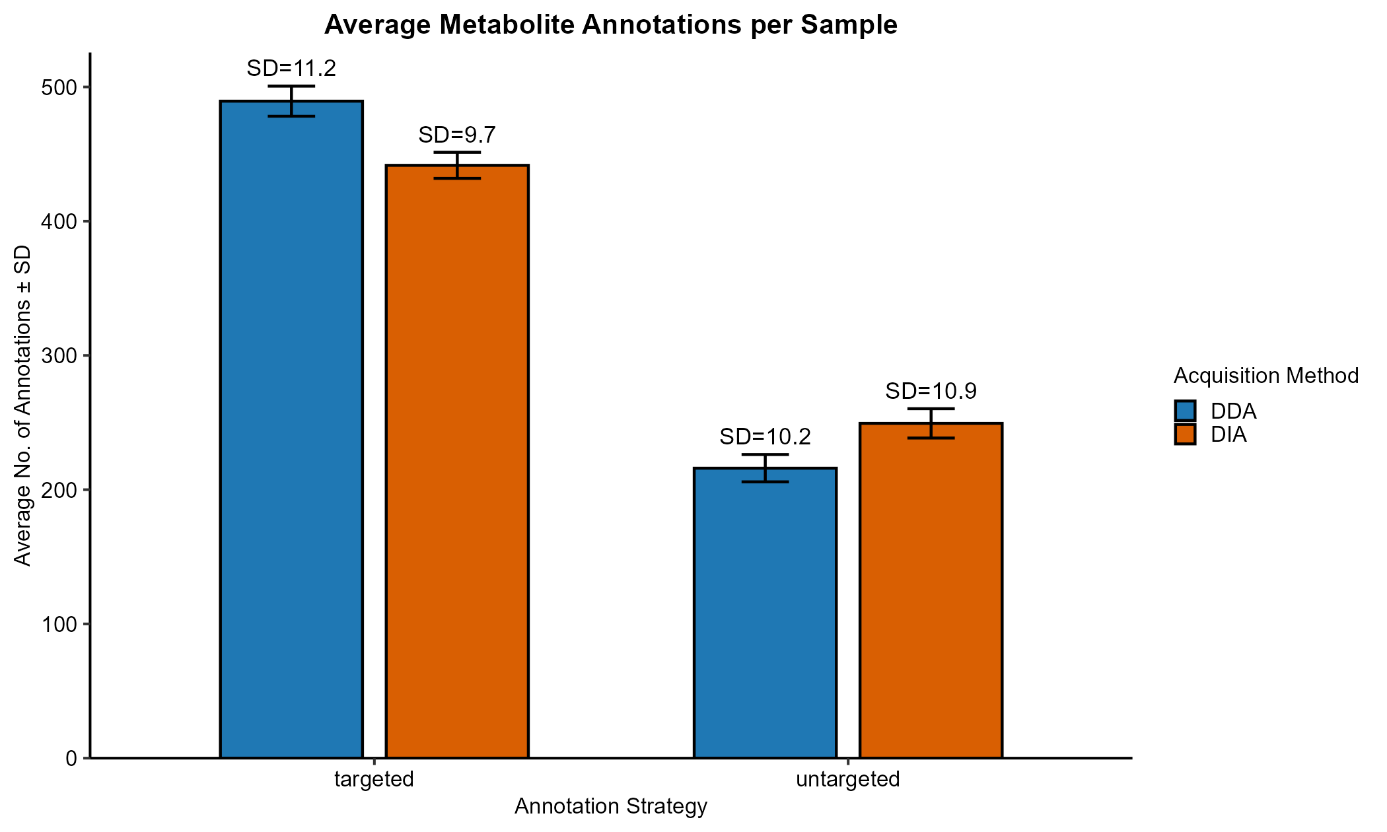


Figure S 7 Barplot of average metabolite annotation counts across five SRM 1950 technical replicate injections using targeted (left) and untargeted (right) processing of DDA (blue) and DIA (orange) data.


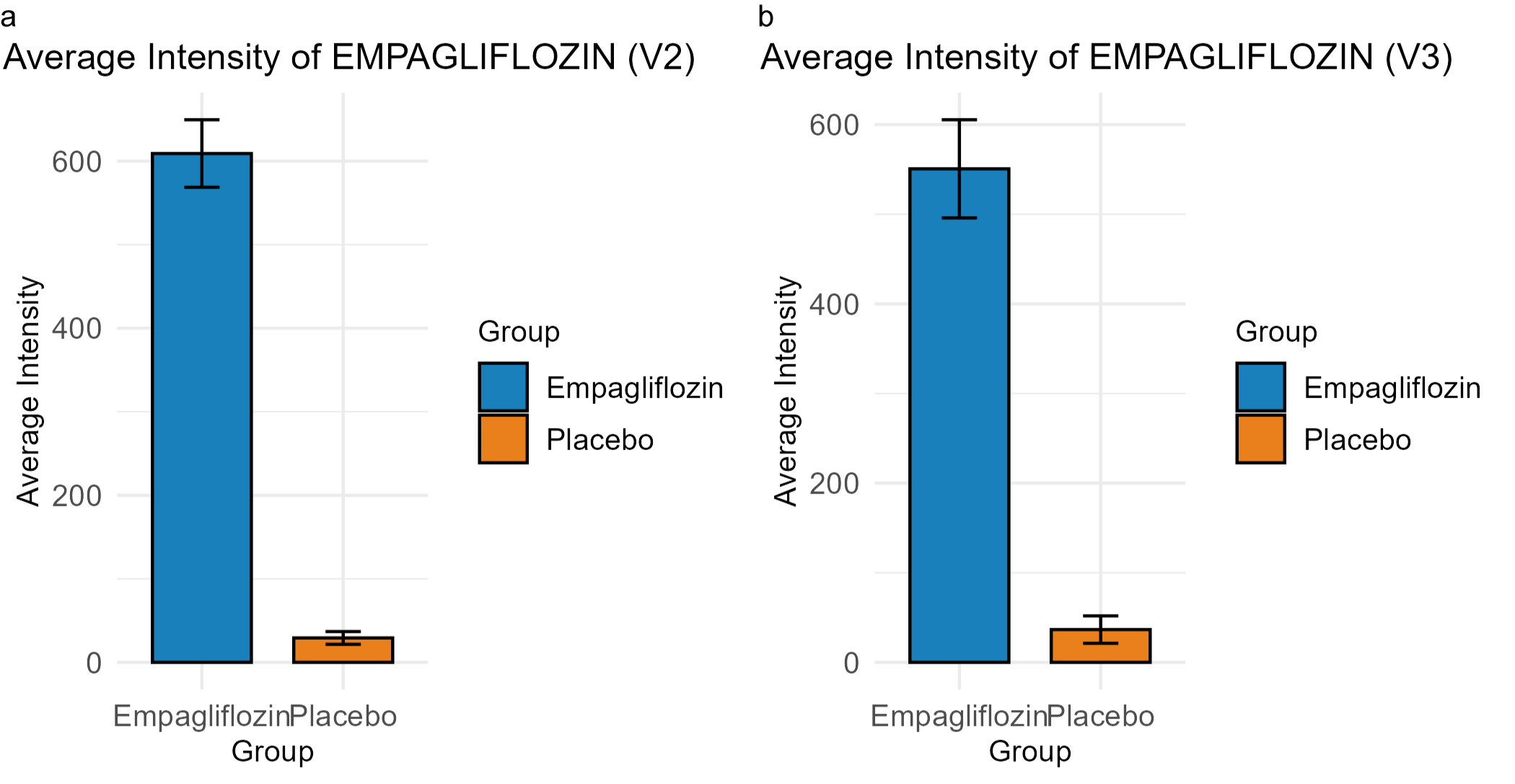


Figure S 8. Bar chart of normalized intensity values of empagliflozin measured in positive ion mode data at V2 (a) and V3 (b), comparing empagliflozin and placebo groups. Error bars represent standard deviation.


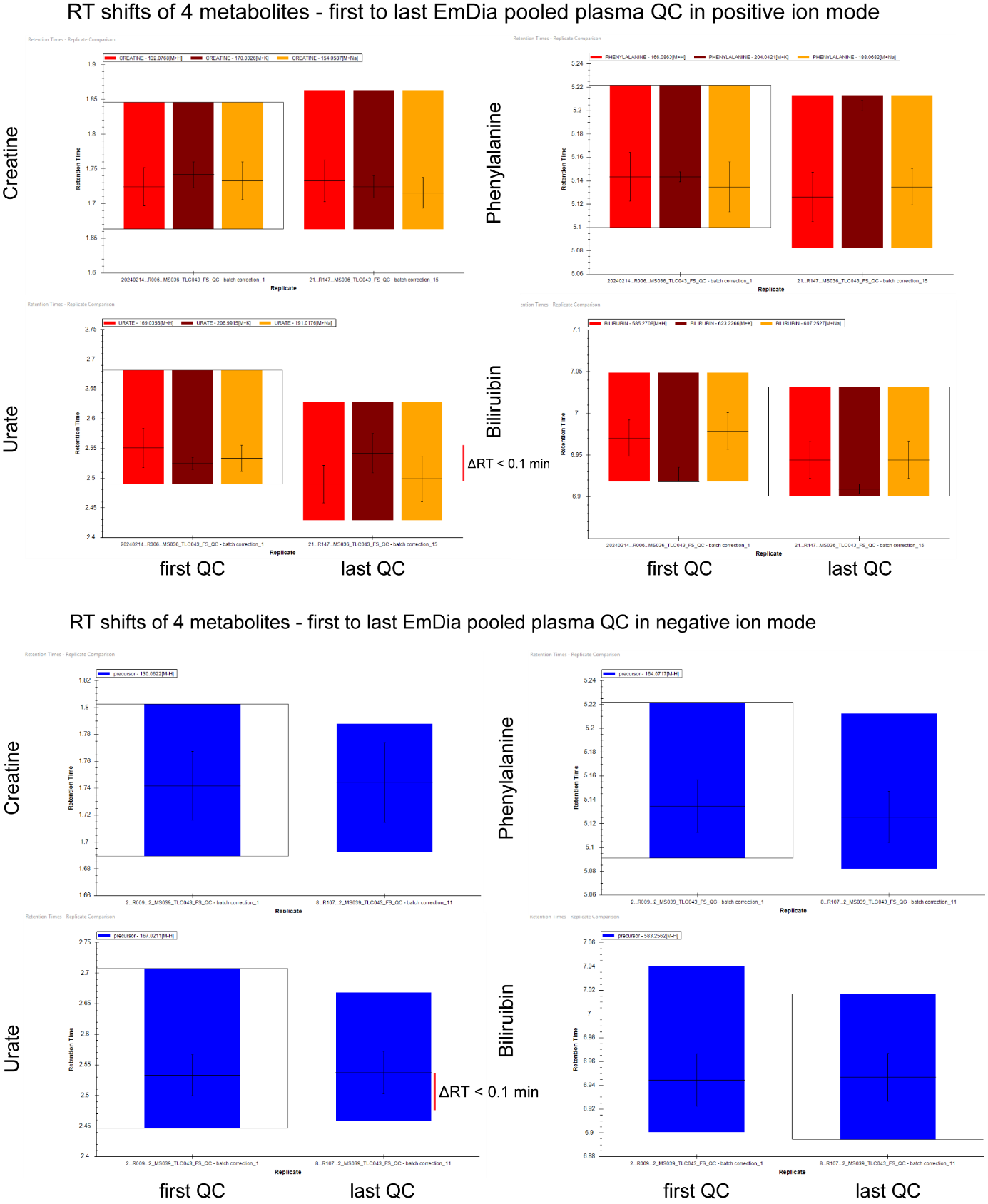


Figure S 9. Chromatographic retention time (RT) stability across batches shown for the selected metabolites creatine, urate, phenylalanine and bilirubin. Integration boundary and peak apex are shown for first and last QC QC injection of the EmDia cohort measurement in positive ion mode (top panels). Analogous data is shown for negative ion mode (bottom). RT deviation remained below 0.1 min over the course of 700 injections on the same Kinetex F5 column in both ion modes.


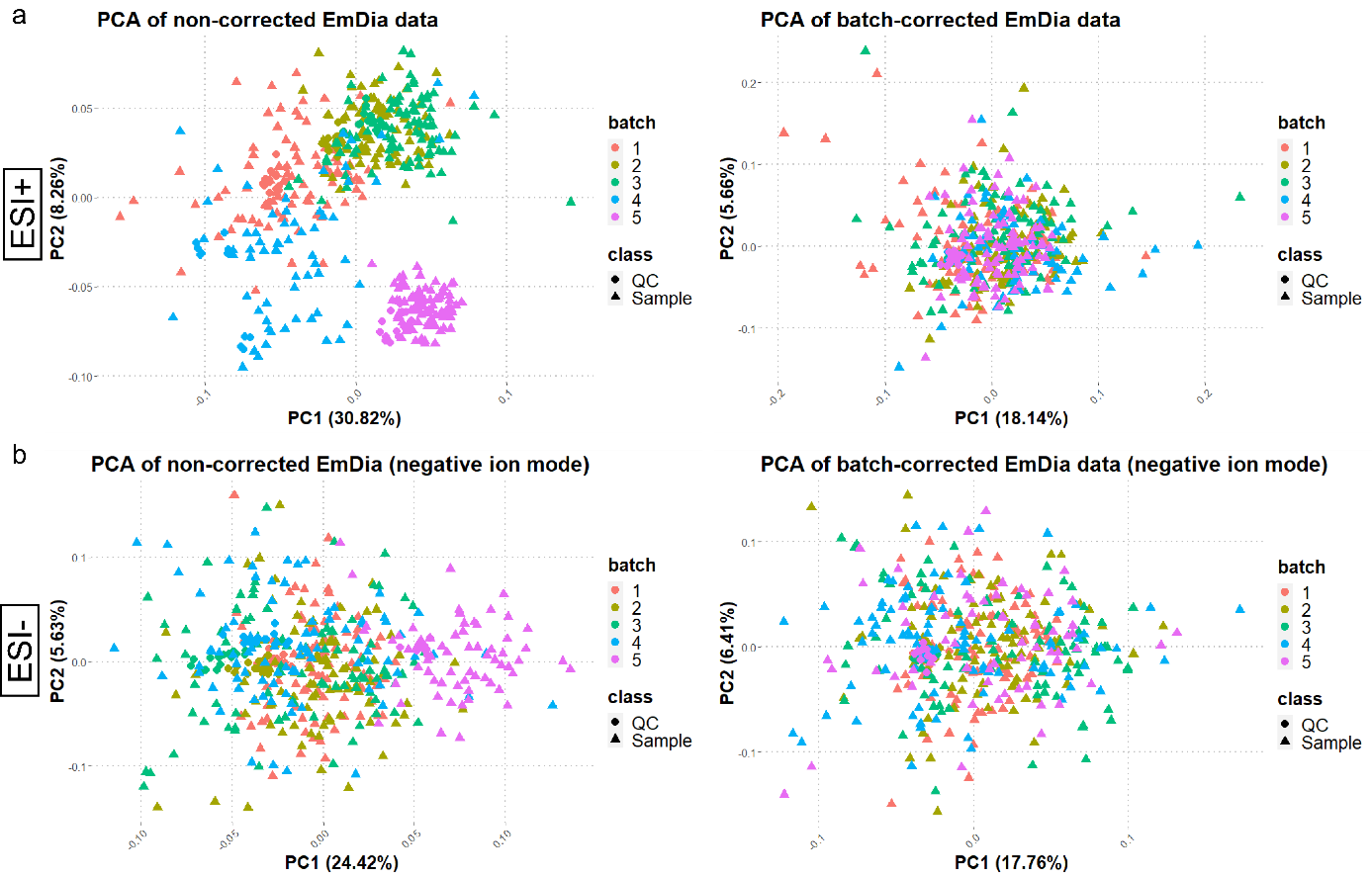


Figure S 10. Batch correction of the EmDia cohort measurement of (a) positive ion mode data and (b) negative ion mode data. Principal component analysis of non-corrected raw data of five batches, including samples and QCs (left panels), PCA of batch-corrected data of five batches including samples and QCs (right panels). Batch correction was performed using the Quality Control-Robust Spline Correction (QC-RSC).^1^

Table S 1. Metabolites used for Skyline transition list for the annotation of metabolites in the EmDia cohort in positive and/or negative ion mode (inclusion indicated in green). ***Added as annotation obtained by untargeted analysis, reference standard not measured yet.

| **Metabolite Name** | **RT** | **CAS** | **HMDB** | **positive ion mode** | **negative ion mode** |
| --- | --- | --- | --- | --- | --- |
| 10-HYDROXYDECANOATE | 6.44 | 1679-53-4 | HMDB0244272 |  |  |
| 1-AMINOCYCLOPROPANECARBOXYLATE | 1.62 | 22059-21-8 | HMDB0036458 |  |  |
| 1-METHYLADENOSINE | 2.73 | 15763-06-1 | HMDB0003331 |  |  |
| 1-METHYL-L-HISTIDINE | 1.49 | 332-80-9 | HMDB0000001 |  |  |
| 1-METHYLNICOTINAMIDE | 1.66 | 3106-60-3 | HMDB0000699 |  |  |
| 2,4-DIHYDROXYACETOPHENONE | 6.44 | 89-84-9 | HMDB0029659 |  |  |
| 2-AMINOISOBUTYRATE | 1.69 | 62-57-7 | HMDB0001906 |  |  |
| 2-AMINOPHENOL | 2.48 | 95-55-6 | - |  |  |
| DEOXY HEXOSE C6H12O5 | 1.74 | 154-17-6 | HMDB0062477 |  |  |
| 2-HYDROXYPYRIDINE | 3.87 | 142-08-5 | HMDB0013751 |  |  |
| 2-METHYLCITRATE | 1.5 | 6061-96-7 | HMDB0000379 |  |  |
| 2-PROPENOATE | 1.6 | 79-10-7 | HMDB0031647 |  |  |
| 3-HYDROXYMETHYLGLUTARATE | 3.14 | 503-49-1 | HMDB0000355 |  |  |
| 3-METHYL-2-OXOVALERATE | 6.6 | 1460-34-0 | HMDB0000491 |  |  |
| 3-METHYLGLUTACONATE | 5.44 | 5746-90-7 | HMDB0000522 |  |  |
| 3,4,5-TRIMETHOXYCINNAMATE*** | 5.96 | 90-50-6 | HMDB0002511 |  |  |
| 4-COUMARATE | 6.01 | 501-98-4 | HMDB0002035 |  |  |
| 4-HYDROXYBENZALDEHYDE | 6.04 | 123-08-0 | HMDB0011718 |  |  |
| 5-HYDROXYINDOLEACETATE | 5.69 | 54-16-0 | HMDB0000763 |  |  |
| 5-METHOXYINDOLEACETATE | 6.3 | 3471-31-6 | HMDB0004096 |  |  |
| 5-METHYLCYTIDINE | 2.61 | 2140-61-6 | HMDB0000982 |  |  |
| 6-PHOSPHOGLUCONATE | 1.63 | 921-62-0 | HMDB0001316 |  |  |
| 7-METHYLXANTHINE*** | 5.09 | 552-62-5 | HMDB0001991 |  |  |
| ADENOSINE 3,5-DIPHOSPHATE | 2 | 1053-73-2 | HMDB0000061 |  |  |
| ADENOSINE-MONOPHOSPHATE | 2.26 | 61-19-8 | HMDB0000045 |  |  |
| ALPHA-D-GLUCOSE (MONOSACCHARIDE C6H12O6) | 1.61 | 492-62-6 | HMDB0003345 |  |  |
| AMINOISOBUTANOATE | 1.71 | 144-90-1 | HMDB0003911 |  |  |
| ARGININE | 1.49 | 74-79-3 | HMDB0000517 |  |  |
| ASCORBATE | 2 | 50-81-7 | HMDB0000044 |  |  |
| ASPARAGINE | 1.56 | 70-47-3 | HMDB0000168 |  |  |
| ASPARTATE | 1.59 | 56-84-8 | HMDB0000191 |  |  |
| BENZYL ALCOHOL | 5.83 | 100-51-6 | HMDB0003119 |  |  |
| BETAINE | 1.72 | 107-43-7 | HMDB0000043 |  |  |
| BILIRUBIN | 6.95 | 635-65-4 | HMDB0000054 |  |  |
| BILIVERDIN | 7.1 | 114-25-0 | HMDB0001008 |  |  |
| CAFFEINE | 5.67 | 58-08-2 | HMDB0001847 |  |  |
| CHENODEOXYCHOLATE | 6.9 | 474-25-9 | HMDB0000518 |  |  |
| CHOLATE | 6.74 | 81-25-4 | HMDB0000619 |  |  |
| CIS-4-HYDROXY-D-PROLINE | 1.59 | 2584-71-6 | HMDB0060460 |  |  |
| CITRAMALATE | 2.98 | 597-44-4 | HMDB0000426 |  |  |
| CITRATE | 2.45 | 77-92-9 | HMDB0000094 |  |  |
| CITRULLINE | 1.62 | 372-75-8 | HMDB0000904 |  |  |
| CORTICOSTERONE | 6.72 | 50-22-6 | HMDB0001547 |  |  |
| CORTISOL | 6.45 | 50-23-7 | HMDB0000063 |  |  |
| CORTISONE | 6.48 | 53-06-5 | HMDB0002802 |  |  |
| CREATINE | 1.72 | 57-00-1 | HMDB0000064 |  |  |
| CREATININE | 1.69 | 60-27-5 | HMDB0000562 |  |  |
| CYSTEINE | 1.62 | 52-90-4 | HMDB0000574 |  |  |
| CYSTINE | 1.53 | 56-89-3 | HMDB0000192 |  |  |
| CYTIDINE | 2.03 | 65-46-3 | HMDB0000089 |  |  |
| DEOXYCARNITINE | 1.82 | 407-64-7 | HMDB0001161 |  |  |
| DEOXYCHOLATE | 6.09 | 83-44-3 | HMDB0000626 |  |  |
| DEOXYGUANOSINE | 5.05 | 961-07-9 | HMDB0000085 |  |  |
| DEOXYURIDINE-MONOPHOSPHATE | 2.46 | 964-26-1 | HMDB0001409 |  |  |
| D-MANNOSAMINE (AMINO SUGAR C6H13NO5) | 1.49 | 579-33-9 | - |  |  |
| DOCOSAHEXAENOATE | 7 | 6217-54-5 | HMDB0002183 |  |  |
| EMPAGLIFLOZIN | 6.53 | 864070-44-0 | HMDB0251778 |  |  |
| GALACTURONATE | 5.06 | 685-73-4 | HMDB0003402 |  |  |
| GAMMA-AMINOBUTYRATE | 1.59 | 56-12-2 | HMDB0000112 |  |  |
| GAMMA-LINOLENATE | 7.62 | 506-26-3 | HMDB0003073 |  |  |
| GLUCONATE | 1.65 | 526-95-4 | HMDB0000625 |  |  |
| GLUCOSE 1-PHOSPHATE (C6H13O9P) | 1.6 | 59-56-3 | HMDB0001586 |  |  |
| GLUTAMATE | 1.61 | 56-86-0 | HMDB0000148 |  |  |
| GLUTAMINE | 1.58 | 56-85-9 | HMDB0000641 |  |  |
| GLUTARYLCARNITINE | 5.08 | 102636-82-8 | HMDB0013130 |  |  |
| GLUTATHIONE REDUCED | 2.33 | 70-18-8 | HMDB0000125 |  |  |
| GLYCERALDEHYDE | 2.26 | 56-82-6 | HMDB0001051 |  |  |
| GLYCERATE | 1.75 | 473-81-4 | HMDB0000139 |  |  |
| GLYCEROL 3-PHOSPHATE | 1.63 | 57-03-4 | HMDB0000126 |  |  |
| GLYCEROPHOSPHOCHOLINE | 1.64 | 28319-77-9 | HMDB0000086 |  |  |
| GLYCINE | 1.55 | 56-40-6 | HMDB0000123 |  |  |
| GLYCOCHENODEOXYCHOLATE | 6.9 | 640-79-9 | HMDB0000637 |  |  |
| GLYCOCHOLATE | 6.54 | 475-31-0 | HMDB0000138 |  |  |
| GUAIACOL | 5.81 | 90-05-1 | HMDB0001398 |  |  |
| GUANIDINOACETATE | 1.61 | 352-97-6 | HMDB0000128 |  |  |
| GUANIDINOSUCCINATE | 1.67 | 6133-30-8 | HMDB0003157 |  |  |
| HIPPURATE | 5.74 | 495-69-2 | HMDB0000714 |  |  |
| HISTIDINE | 1.47 | 71-00-1 | HMDB0000177 |  |  |
| HOMOCYSTEINE | 1.79 | 454-29-5 | HMDB0000742 |  |  |
| HYDROXYPHENYLLACTATE | 5.45 | 306-23-0 | HMDB0000755 |  |  |
| HYPOXANTHINE | 2.72 | 68-94-0 | HMDB0000157 |  |  |
| INDOLE | 7.39 | 120-72-9 | HMDB0000738 |  |  |
| INDOLE-3-ACETATE | 6.39 | 87-51-4 | HMDB0000197 |  |  |
| INDOLE-3-METHYL ACETATE | 7 | 1912-33-0 | HMDB0029738 |  |  |
| INDOLE-3-PYRUVATE | 6.13 | 392-12-1 | HMDB0060484 |  |  |
| INDOLEACETALDEHYDE | 5.45 | 2591-98-2 | HMDB0001190 |  |  |
| INDOXYL SULFATE | 5.64 | 487-94-5 | HMDB0000682 |  |  |
| INOSINE-MONOPHOSPHATE | 2.41 | 131-99-7 | HMDB0000175 |  |  |
| ISOCITRATE | 2 | 320-77-4 | HMDB0000193 |  |  |
| ISOLEUCINE | 2.84 | 73-32-5 | HMDB0000172 |  |  |
| ITACONATE | 1.6 | 97-65-4 | HMDB0002092 |  |  |
| KETOLEUCINE | 5.8 | 816-66-0 | HMDB0000695 |  |  |
| KYNURENATE | 5.66 | 492-27-3 | HMDB0000715 |  |  |
| KYNURENINE | 5.13 | 2922-83-0 | HMDB0000684 |  |  |
| LACTOSE (DISACCHARIDE C12H22O11) | 1.64 | 63-42-3 | HMDB0000186 |  |  |
| L-ALANINE | 1.58 | 56-41-7 | HMDB0000161 |  |  |
| LAURATE | 7.89 | 143-07-7 | HMDB0000638 |  |  |
| L-CARNITINE | 1.71 | 541-15-1 | HMDB0000062 |  |  |
| LEUCINE | 3.11 | 61-90-5 | HMDB0000687 |  |  |
| L-GULONOLACTONE | 1.67 | 1615286 | - |  |  |
| LITHOCHOLYLTAURINE | 6.74 | 516-90-5 | HMDB0000722 |  |  |
| L-ORNITHINE | 1.43 | 3184-13-2 | HMDB0000214 |  |  |
| LUMICHROME | 6.17 | 1086-80-2 | HMDB0254199 |  |  |
| LYSINE | 1.44 | 56-87-1 | HMDB0000182 |  |  |
| METFORMIN | 1.8 | 657-24-9 | HMDB0001921 |  |  |
| METHIONINE | 2.25 | 63-68-3 | HMDB0000696 |  |  |
| METHIONINE SULFOXIMINE | 1.57 | 15985-39-4 | - |  |  |
| METHYL JASMONATE | 7.37 | 39924-52-2 | HMDB0036583 |  |  |
| METHYLMALONATE | 3.16 | 516-05-2 | HMDB0000202 |  |  |
| N,N,N-TRIMETHYLLYSINE | 1.52 | 19253-88-4 | HMDB0001325 |  |  |
| N,N-DIMETHYLARGININE | 1.73 | 30315-93-6 | HMDB0001539 |  |  |
| N6--ADENINE | 5.79 | 2365-40-4 | HMDB0245646 |  |  |
| N8-ACETYLSPERMIDINE | 1.51 | 13431-24-8 | HMDB0002189 |  |  |
| N-ACETYL CARNOSINE | 2.35 | 56353-15-2 | HMDB0012881 |  |  |
| N-ACETYLALANINE | 3.06 | 97-69-8 | HMDB0000766 |  |  |
| N-ACETYLNEURAMINATE | 1.73 | 131-48-6 | HMDB0000230 |  |  |
| N-ACETYLPHENYLALANINE | 6.02 | 2018-61-3 | HMDB0000512 |  |  |
| N-ACETYLSEROTONIN | 5.74 | 1210-83-9 | HMDB0001238 |  |  |
| N-ACETYLTRYPTOPHAN | 6.11 | 1218-34-4 | HMDB0013713 |  |  |
| N-FORMYL-L-METHIONINE | 5.39 | 4289-98-9 | HMDB0001015 |  |  |
| N-GLYCOLYLNEURAMINIC ACID | 1.67 | 1113-83-3 | HMDB0000833 |  |  |
| NICOTINAMIDE | 2.54 | 98-92-0 | HMDB0001406 |  |  |
| N-METHYLARGININE | 1.55 | 17035-90-4 | HMDB0250980 |  |  |
| N-METHYLGLUTAMATE | 1.69 | 6753-62-4 | HMDB0062660 |  |  |
| N-METHYL-2-PYRIDONE-5-CARBOXAMIDE | 5.05 | 701-44-0 | HMDB0004193 |  |  |
| N-METHYL-ALANINE | 1.66 | 3913-67-5 | HMDB0094692 |  |  |
| O-ACETYLCARNITINE | 2.93 | 3040-38-8 | HMDB0000201 |  |  |
| O-ACETYLSERINE | 1.74 | 16354-58-8 | HMDB0002931 |  |  |
| OXALOMALATE | 1.5 | 3687-15-8 | HMDB0245962 |  |  |
| OXOGLUTARATE | 2.58 | 328-50-7 | HMDB0000208 |  |  |
| PALMITOLEATE | 7.56 | 373-49-9 | HMDB0003229 |  |  |
| PANTOTHENATE | 5.18 | 79-83-4 | HMDB0000210 |  |  |
| PARAXANTHINE | 5.4 | 611-59-6 | HMDB0001860 |  |  |
| PHENYLACETALDEHYDE | 6.39 | 122-78-1 | HMDB0006236 |  |  |
| PHENYLALANINE | 5.12 | 63-91-2 | HMDB0000159 |  |  |
| PHENYLETHANOLAMINE | 4.33 | 7568-93-6 | HMDB0001065 |  |  |
| PHOSPHOCREATINE | 1.71 | 67-07-2 | HMDB0001511 |  |  |
| PHOSPHORYLCHOLINE | 1.6 | 645-84-1 | HMDB0001565 |  |  |
| PHYLLOQUINONE | 7.8 | 84-80-0 | HMDB0015157 |  |  |
| PIPECOLATE | 2.17 | 3105-95-1 | HMDB0000716 |  |  |
| PREGNENOLONE SULFATE | 6.81 | 1247-64-9 | HMDB0000774 |  |  |
| PROLINE | 1.75 | 147-85-3 | HMDB0000162 |  |  |
| PYRIDOXAMINE | 1.54 | 85-87-0 | HMDB0001431 |  |  |
| PYROCATECHOL | 5.4 | 120-80-9 | HMDB0000957 |  |  |
| PYROGLUTAMATE | 2.67 | 98-79-3 | HMDB0000267 |  |  |
| QUINATE | 1.76 | 77-95-2 | HMDB0003072 |  |  |
| QUINOLINATE | 2.57 | 89-00-9 | HMDB0000232 |  |  |
| QUINOLINE | 5.29 | 91-22-5 | HMDB0033731 |  |  |
| RAFFINOSE | 1.78 | 512-69-6 | HMDB0003213 |  |  |
| RIBOSE (ALDOPENTOSES C5H10O5) | 1.65 | 50-69-1 | - |  |  |
| SACCHARATE | 1.67 | 87-73-0 | HMDB0029881 |  |  |
| S-ADENOSYLHOMOCYSTEINE | 3.07 | 979-92-0 | HMDB0000939 |  |  |
| SARCOSINE | 1.59 | 107-97-1 | HMDB0000271 |  |  |
| SERINE | 1.55 | 56-45-1 | HMDB0000187 |  |  |
| SEROTONIN | 5.13 | 50-67-9 | HMDB0000259 |  |  |
| SORBITOL | 1.63 | 50-70-4 | HMDB0000247 |  |  |
| TAURINE | 1.57 | 107-35-7 | HMDB0000251 |  |  |
| THEOBROMINE | 5.28 | 83-67-0 | HMDB0002825 |  |  |
| THEOPHYLLINE | 5.41 | 58-55-9 | HMDB0001889 |  |  |
| THREONINE | 1.59 | 72-19-5 | HMDB0000167 |  |  |
| THYROXINE | 6.53 | 51-48-9 | HMDB0000248 |  |  |
| TRANS-ACONITATE | 2.45 | 4023-65-8 | HMDB0000958 |  |  |
| TRANS-CINNAMATE | 6.62 | 140-10-3 | HMDB0000930 |  |  |
| TRIGONELLINE | 1.82 | 535-83-1 | HMDB0000875 |  |  |
| TRIMETHYLAMINE N-OXIDE | 1.69 | 1184-78-7 | HMDB0000925 |  |  |
| TRYPTAMINE | 5.65 | 61-54-1 | HMDB0000303 |  |  |
| TRYPTOPHAN | 5.41 | 153-94-6 | HMDB0013609 |  |  |
| TYRAMINE | 3.55 | 51-67-2 | HMDB0000306 |  |  |
| TYROSINE | 3.08 | 60-18-4 | HMDB0000158 |  |  |
| URACIL | 2.34 | 66-22-8 | HMDB0000300 |  |  |
| URATE | 2.47 | 69-93-2 | HMDB0000289 |  |  |
| URIDINE | 2.87 | 58-96-8 | HMDB0000296 |  |  |
| UROCANATE | 2.4 | 104-98-3 | HMDB0000301 |  |  |
| URSODEOXYCHOLATE | 7.44 | 128-13-2 | HMDB0000946 |  |  |
| VALINE | 1.99 | 72-18-4 | HMDB0000883 |  |  |
| XANTHINE | 2.86 | 69-89-6 | HMDB0000292 |  |  |
| XANTHOSINE | 5.05 | 146-80-5 | HMDB0000299 |  |  |

Table S 2. Log_2_ fold change of annotated metabolites from positive ion mode data comparing placebo and empagliflozin groups using time points V2-V3. Significance is indicated using FDR adjusted *p*-values (**** *p* < 0.0001 | *** *p* < 0.001 | ** *p* < 0.01 | * *p* < 0.05).

| **Metabolite** | **log_2_ fold change** | **FDR adj. *p*-value** | **significance** |
| --- | --- | --- | --- |
| 1-AMINOCYCLOPROPANECARBOXYLATE | -0.087747228 | 5.76E-02 |  |
| 1-METHYLADENOSINE | -0.029053064 | 6.83E-01 |  |
| 1-METHYL-L-HISTIDINE | -0.042952165 | 6.78E-01 |  |
| 1-METHYLNICOTINAMIDE | -0.141383102 | 1.66E-01 |  |
| 2-AMINOISOBUTYRATE | -0.204729472 | 2.24E-04 | *** |
| 2-AMINOPHENOL | 0.098963494 | 1.06E-01 |  |
| DEOXYHEXOSE | 1.769507468 | 1.78E-26 | **** |
| 2-HYDROXYPYRIDINE | 0.016298385 | 9.45E-01 |  |
| 2-METHYLCITRATE | -0.077571727 | 5.05E-02 |  |
| 3-HYDROXYMETHYLGLUTARATE | 0.114608474 | 2.23E-01 |  |
| 3-METHYLGLUTACONATE | 0.097107023 | 2.42E-01 |  |
| 4-COUMARATE | -0.002919711 | 9.87E-01 |  |
| 3,4,5-TRIMETHOXYCINNAMATE | -0.71120698 | 1.09E-02 | * |
| 4-HYDROXYBENZALDEHYDE | -0.011355294 | 9.35E-01 |  |
| 5-HYDROXYINDOLEACETATE | 0.06307277 | 4.73E-01 |  |
| 5-METHOXYINDOLEACETATE | 0.01346181 | 9.35E-01 |  |
| 6-PHOSPHOGLUCONATE | -0.061965449 | 2.42E-01 |  |
| 7-METHYLXANTHINE | -0.482161634 | 6.39E-02 |  |
| ADENOSINE-MONOPHOSPHATE | -0.088540857 | 5.47E-01 |  |
| AMINO SUGAR C6H13NO5 | 0.065438081 | 2.25E-01 |  |
| ARGININE | -0.018503539 | 8.80E-01 |  |
| ASPARAGINE | -0.017222936 | 7.84E-01 |  |
| ASPARTATE | 0.00377875 | 9.68E-01 |  |
| BETAINE | -0.034144298 | 6.83E-01 |  |
| BILIRUBIN | 0.033128542 | 8.91E-01 |  |
| BILIVERDIN | -0.014913675 | 9.35E-01 |  |
| CAFFEINE | -0.150239683 | 6.30E-01 |  |
| CHENODEOXYCHOLATE | -0.00268658 | 9.87E-01 |  |
| CIS-4-HYDROXY-D-PROLINE | -0.048653917 | 6.82E-01 |  |
| CITRATE | -0.238201202 | 1.69E-03 | ** |
| CITRULLINE | -0.120135755 | 3.07E-01 |  |
| CORTICOSTERONE | -0.235854511 | 5.59E-01 |  |
| CORTISOL | -0.260973409 | 1.28E-03 | ** |
| CORTISONE | -0.124480132 | 6.39E-02 |  |
| CREATINE | -0.232120176 | 2.18E-01 |  |
| CREATININE | -0.157898221 | 2.05E-02 | * |
| CYSTINE | -0.03543885 | 6.82E-01 |  |
| DEOXYCARNITINE | -0.121376478 | 2.34E-01 |  |
| DEOXYGUANOSINE | -0.800451062 | 4.84E-01 |  |
| DEOXYURIDINE-MONOPHOSPHATE | 0.001565833 | 9.87E-01 |  |
| DISACCHARIDE C12H22O11 | -0.116220642 | 5.04E-01 |  |
| DOCOSAHEXAENOATE | -0.083923886 | 4.66E-01 |  |
| EMPAGLIFLOZIN | -4.381348472 | 6.95E-27 | **** |
| GAMMA-AMINOBUTYRATE | -0.232000981 | 2.58E-05 | **** |
| GAMMA-LINOLENATE | -0.052338763 | 6.55E-01 |  |
| GLUCOSE 1-PHOSPHATE | -0.041918537 | 5.47E-01 |  |
| GLUTAMATE | 0.077610661 | 4.66E-01 |  |
| GLUTAMINE | 0.022651765 | 5.78E-01 |  |
| GLUTARYLCARNITINE | -0.201631373 | 2.77E-01 |  |
| GLUTATHIONE REDUCED | -0.098882647 | 3.76E-01 |  |
| GLYCEROPHOSPHOCHOLINE | 0.110504255 | 1.06E-01 |  |
| GLYCINE | -0.006063074 | 9.68E-01 |  |
| GLYCOCHENODEOXYCHOLATE | -0.267118434 | 4.53E-01 |  |
| GLYCOCHOLATE | -0.841729506 | 3.07E-01 |  |
| GUANIDINOACETATE | 0.071910141 | 6.11E-01 |  |
| GUANIDINOSUCCINATE | -0.416006297 | 2.01E-02 | * |
| HIPPURATE | -0.177194988 | 5.47E-01 |  |
| HISTIDINE | -0.024750913 | 5.17E-01 |  |
| HOMOCYSTEINE | -0.03865025 | 5.47E-01 |  |
| HYPOXANTHINE | -0.201121548 | 1.66E-01 |  |
| INDOLE | 0.008621074 | 9.35E-01 |  |
| INDOLE-3-ACETATE | -0.240369625 | 4.42E-01 |  |
| INDOLE-3-METHYL ACETATE | -0.120892884 | 5.48E-01 |  |
| INDOLE-3-PYRUVATE | -0.012676954 | 9.35E-01 |  |
| INDOLEACETALDEHYDE | 0.010106794 | 9.34E-01 |  |
| INOSINE-MONOPHOSPHATE | -0.302772181 | 1.58E-01 |  |
| ISOCITRATE | -0.093302348 | 3.84E-01 |  |
| ISOLEUCINE | -0.17250436 | 5.82E-03 | ** |
| KYNURENATE | -0.001121028 | 9.89E-01 |  |
| KYNURENINE | 0.022450511 | 7.92E-01 |  |
| L-ALANINE | 0.001010335 | 9.87E-01 |  |
| L-CARNITINE | -0.056328886 | 3.35E-01 |  |
| LEUCINE | -0.148953265 | 1.23E-02 | * |
| L-ORNITHINE | -0.056584741 | 2.62E-01 |  |
| LUMICHROME | -0.052423895 | 7.38E-01 |  |
| LYSINE | -0.027341161 | 5.47E-01 |  |
| METFORMIN | 0.226064774 | 4.47E-01 |  |
| METHIONINE | -0.135816873 | 2.92E-02 | * |
| METHYL JASMONATE | -0.004076424 | 9.42E-01 |  |
| MONOSACCHARIDE C6H12O6 | 0.083720818 | 1.66E-01 |  |
| N,N,N-TRIMETHYLLYSINE | -0.049725891 | 7.84E-01 |  |
| N,N-DIMETHYLARGININE | -0.089111449 | 6.78E-02 |  |
| N6--ADENINE | -0.304202658 | 4.82E-06 | **** |
| N8-ACETYLSPERMIDINE | -0.114011941 | 3.06E-01 |  |
| N-ACETYL CARNOSINE | -0.10814068 | 3.07E-01 |  |
| N-ACETYLALANINE | -0.054365572 | 1.15E-01 |  |
| N-ACETYLNEURAMINATE | -0.123366488 | 1.06E-01 |  |
| N-ACETYLSEROTONIN | -0.174546987 | 1.67E-01 |  |
| N-ACETYLTRYPTOPHAN | -0.240574417 | 2.02E-01 |  |
| NICOTINAMIDE | -0.085501803 | 4.53E-01 |  |
| N-METHYLARGININE | 0.12658037 | 1.33E-01 |  |
| O-ACETYLCARNITINE | -0.190634866 | 5.20E-01 |  |
| OXALOMALATE | -0.015788171 | 8.71E-01 |  |
| OXOGLUTARATE | -0.081547984 | 1.66E-01 |  |
| PALMITOLEATE | -0.10252821 | 3.40E-01 |  |
| PANTOTHENATE | 0.090275345 | 6.79E-01 |  |
| PARAXANTHINE | -0.149316039 | 5.47E-01 |  |
| PHENYLACETALDEHYDE | 0.031866823 | 8.58E-01 |  |
| PHENYLALANINE | -0.068777536 | 2.13E-01 |  |
| PHENYLETHANOLAMINE | 0.003192991 | 9.87E-01 |  |
| PHOSPHOCREATINE | -0.111642156 | 1.53E-01 |  |
| PHOSPHORYLCHOLINE | -0.05656829 | 4.15E-01 |  |
| PHYLLOQUINONE | 0.057844477 | 2.02E-01 |  |
| PIPECOLATE | -0.054505304 | 7.85E-01 |  |
| PROLINE | -0.110973971 | 2.67E-01 |  |
| PYRIDOXAMINE | -0.031572437 | 5.00E-01 |  |
| PYROGLUTAMATE | 0.028729181 | 4.66E-01 |  |
| QUINATE | -0.074815327 | 7.84E-01 |  |
| QUINOLINATE | -0.003398422 | 9.87E-01 |  |
| QUINOLINE | 0.020911425 | 7.21E-01 |  |
| RAFFINOSE | -0.164228126 | 2.88E-01 |  |
| RIBOSE | 0.01078349 | 9.52E-01 |  |
| SACCHARATE | 0.012375173 | 6.91E-01 |  |
| S-ADENOSYLHOMOCYSTEINE | 0.033246684 | 7.87E-01 |  |
| SARCOSINE | -0.138192973 | 2.76E-01 |  |
| SERINE | 0.062580938 | 4.04E-01 |  |
| SEROTONIN | -0.21106234 | 8.66E-01 |  |
| TAURINE | -0.0772402 | 1.62E-01 |  |
| THEOBROMINE | -0.281822647 | 3.37E-01 |  |
| THEOPHYLLINE | -0.151602929 | 5.47E-01 |  |
| THREONINE | 0.018711548 | 7.87E-01 |  |
| THYROXINE | 0.02292247 | 7.85E-01 |  |
| TRANS-ACONITATE | -0.186153127 | 1.75E-02 | * |
| TRANS-CINNAMATE | -0.078648438 | 1.66E-01 |  |
| TRIGONELLINE | -0.004372683 | 9.87E-01 |  |
| TRIMETHYLAMINE N-OXIDE | -0.190387983 | 1.06E-01 |  |
| TRYPTOPHAN | 0.031046318 | 6.83E-01 |  |
| TYROSINE | -0.010421938 | 9.36E-01 |  |
| URACIL | 0.050148888 | 5.49E-01 |  |
| URATE | 0.219616732 | 1.09E-04 | *** |
| URIDINE | 0.018178562 | 8.78E-01 |  |
| UROCANATE | 0.064646281 | 6.00E-01 |  |
| VALINE | -0.102656747 | 4.06E-02 | * |
| XANTHINE | -0.325589977 | 5.47E-01 |  |

Table S 3 Log_2_ fold change of annotated metabolites from negative ion mode data comparing placebo and empagliflozin groups using time points V2-V3. Significance is indicated using FDR adjusted *p*-values (**** *p* < 0.0001 | *** *p* < 0.001 | ** *p* < 0.01 | * *p* < 0.05).

| **Metabolite** | **log_2_ fold change** | **FDR adj. *p*-value** | **significance** |
| --- | --- | --- | --- |
| 10-HYDROXYDECANOATE | -0.25635478 | 3.77E-03 | ** |
| 1-METHYL-L-HISTIDINE | -0.29487339 | 5.14E-01 |  |
| 2,4-DIHYDROXYACETOPHENONE | 0.239035298 | 6.77E-01 |  |
| DEOXYHEXOSE | 2.43453972 | 2.80E-24 | **** |
| 2-PROPENOATE | 0.103453028 | 9.74E-02 |  |
| 3-HYDROXYMETHYLGLUTARATE | -0.029894514 | 8.29E-01 |  |
| 3-METHYL-2-OXOVALERATE | -0.124814616 | 1.49E-01 |  |
| 3-METHYLGLUTACONATE | 0.00193009 | 9.94E-01 |  |
| 4-COUMARATE | 0.001977438 | 9.94E-01 |  |
| 5-HYDROXYINDOLEACETATE | 0.073207818 | 4.08E-01 |  |
| 5-METHOXYINDOLEACETATE | 0.010036005 | 9.50E-01 |  |
| 5-METHYLCYTIDINE | -0.036275119 | 7.31E-01 |  |
| ADENOSINE 3,5-DIPHOSPHATE | 0.024596182 | 8.94E-01 |  |
| ADENOSINE-MONOPHOSPHATE | -0.074056945 | 6.77E-01 |  |
| ALPHA-D-GLUCOSE | -0.046438818 | 8.53E-01 |  |
| AMINOISOBUTANOATE | 0.021355211 | 8.72E-01 |  |
| ARGININE | -0.038280559 | 7.24E-01 |  |
| ASCORBATE | -0.010850334 | 9.13E-01 |  |
| ASPARAGINE | -0.068849685 | 1.74E-01 |  |
| ASPARTATE | -0.073841641 | 6.59E-02 |  |
| BENZYL ALCOHOL | -0.110341031 | 6.77E-01 |  |
| BILIRUBIN | 0.015902498 | 9.55E-01 |  |
| BILIVERDIN | -0.023521664 | 8.53E-01 |  |
| CELLOBIOSE | -0.717266923 | 3.03E-01 |  |
| CHENODEOXYCHOLATE | -0.104217631 | 7.36E-01 |  |
| CHOLATE | 0.508432125 | 4.45E-01 |  |
| CITRAMALATE | 0.09409376 | 2.19E-01 |  |
| CITRATE | -0.18556318 | 3.21E-03 | ** |
| CITRULLINE | -0.192677123 | 1.25E-01 |  |
| CORTISOL | -0.311253259 | 3.21E-03 | ** |
| CORTISONE | -0.11089064 | 1.92E-01 |  |
| CREATINE | -0.173159745 | 4.26E-01 |  |
| CREATININE | -0.153434818 | 7.83E-02 |  |
| CYSTEINE | -0.099213364 | 1.46E-01 |  |
| CYSTINE | -0.146012953 | 1.20E-03 | ** |
| CYTIDINE | 0.081153623 | 1.69E-01 |  |
| DEOXYCHOLATE | -0.301462282 | 1.63E-01 |  |
| DEOXYURIDINE-MONOPHOSPHATE | -0.016748923 | 8.33E-01 |  |
| EMPAGLIFLOZIN | -0.632515411 | 1.41E-03 | ** |
| GALACTURONATE | -0.048098565 | 8.31E-01 |  |
| GLUCONATE | -0.015222583 | 9.06E-01 |  |
| GLUCOSE 1-PHOSPHATE | -0.145672633 | 4.01E-01 |  |
| GLUTAMATE | 0.052769572 | 7.22E-01 |  |
| GLUTAMINE | -0.013791825 | 8.33E-01 |  |
| GLYCERALDEHYDE | -0.052843147 | 5.02E-01 |  |
| GLYCERATE | -0.0652281 | 4.45E-01 |  |
| GLYCEROL 3-PHOSPHATE | -0.158445988 | 1.64E-02 | * |
| GLYCINE | -0.056407412 | 4.45E-01 |  |
| GLYCOCHENODEOXYCHOLATE | -0.310057788 | 4.52E-01 |  |
| GLYCOCHOLATE | -0.747594781 | 3.79E-01 |  |
| GUAIACOL | -0.108908498 | 8.31E-01 |  |
| GUANIDINOSUCCINATE | -0.376238018 | 5.27E-02 |  |
| HIPPURATE | -0.115432091 | 7.36E-01 |  |
| HISTIDINE | -0.089474852 | 7.87E-02 |  |
| HYDROXYPHENYLLACTATE | 0.094166549 | 4.45E-01 |  |
| HYPOXANTHINE | -0.179603538 | 2.34E-01 |  |
| INDOLE | 0.011004925 | 8.94E-01 |  |
| INDOLE-3-ACETATE | -0.219932947 | 4.45E-01 |  |
| INDOLE-3-METHYL ACETATE | -0.142807754 | 5.14E-01 |  |
| INDOLE-3-PYRUVATE | -0.001245231 | 9.97E-01 |  |
| INDOXYL SULFATE | 0.066220488 | 8.12E-01 |  |
| INOSINE-MONOPHOSPHATE | -0.250300277 | 1.49E-01 |  |
| ISOCITRATE | -0.154144119 | 1.64E-02 | * |
| ISOLEUCINE | -0.163287432 | 2.59E-02 | * |
| ITACONATE | 0.044939355 | 5.56E-01 |  |
| KETOLEUCINE | -0.125397737 | 1.49E-01 |  |
| KYNURENINE | 0.034512888 | 6.77E-01 |  |
| L-ALANINE | -0.031700713 | 7.36E-01 |  |
| LAURATE | -0.319550274 | 9.53E-03 | ** |
| LEUCINE | -0.152409495 | 1.64E-02 | * |
| L-GULONOLACTONE | 0.074547387 | 3.71E-01 |  |
| LITHOCHOLYLTAURINE | -0.537610131 | 6.59E-02 |  |
| L-ORNITHINE | -0.194865688 | 7.83E-02 |  |
| LYSINE | -0.025841054 | 6.49E-01 |  |
| METHIONINE | -0.047245968 | 5.21E-01 |  |
| METHIONINE SULFOXIMINE | -0.052976346 | 8.06E-01 |  |
| METHYL JASMONATE | -0.040471444 | 8.33E-01 |  |
| METHYLMALONATE | -0.121412925 | 1.69E-01 |  |
| N,N-DIMETHYLARGININE | -0.115584762 | 9.56E-02 |  |
| N-ACETYL CARNOSINE | -0.091036562 | 4.92E-01 |  |
| N-ACETYLALANINE | 0.039450326 | 8.12E-01 |  |
| N-ACETYLNEURAMINATE | -0.25487628 | 9.74E-02 |  |
| N-ACETYLPHENYLALANINE | 0.182695773 | 2.02E-01 |  |
| N-ACETYLTRYPTOPHAN | 0.248306016 | 9.74E-02 |  |
| N-FORMYL-L-METHIONINE | -0.029094745 | 8.31E-01 |  |
| N-GLYCOLYLNEURAMINIC ACID | -0.242872433 | 1.44E-01 |  |
| N-METHYLGLUTAMATE | -0.191048241 | 6.52E-02 |  |
| N-METYL-ALANINE | 0.021351479 | 8.72E-01 |  |
| O-ACETYLSERINE | 0.052710443 | 7.22E-01 |  |
| OXOGLUTARATE | -0.093421153 | 2.34E-01 |  |
| PANTOTHENATE | 0.141613069 | 6.07E-01 |  |
| PARAXANTHINE | -0.146606672 | 4.76E-01 |  |
| PHENYLACETALDEHYDE | 0.195472032 | 4.45E-01 |  |
| PHENYLALANINE | -0.041820176 | 5.24E-01 |  |
| PIPECOLATE | -0.04548483 | 8.36E-01 |  |
| PREGNENOLONE SULFATE | 0.152543821 | 3.62E-01 |  |
| PROLINE | -0.057236402 | 5.46E-01 |  |
| PYROCATECHOL | -0.081985915 | 8.04E-01 |  |
| PYROGLUTAMATE | 0.015627696 | 8.23E-01 |  |
| QUINATE | -0.241295428 | 4.45E-01 |  |
| SACCHARATE | 0.015761873 | 8.76E-01 |  |
| SARCOSINE | -0.142880656 | 4.45E-01 |  |
| SERINE | -0.000128187 | 9.98E-01 |  |
| SORBITOL | -0.166650232 | 7.68E-01 |  |
| TAURINE | -0.116889479 | 5.27E-02 |  |
| THEOPHYLLINE | -0.160901925 | 4.45E-01 |  |
| THEOBROMINE | -0.152243473 | 5.74E-01 |  |
| THREONINE | -0.008716124 | 9.43E-01 |  |
| THYROXINE | -0.020628358 | 8.31E-01 |  |
| TRANS-ACONITATE | -0.18999467 | 3.21E-03 | ** |
| TRYPTAMINE | -0.002177034 | 9.94E-01 |  |
| TRYPTOPHAN | 0.01457656 | 8.67E-01 |  |
| TYRAMINE | 0.015491767 | 8.89E-01 |  |
| TYROSINE | -0.00147871 | 9.94E-01 |  |
| URATE | 0.261570146 | 1.60E-06 | **** |
| URIDINE | -0.012236093 | 8.94E-01 |  |
| URSODEOXYCHOLATE | -0.181856306 | 8.19E-01 |  |
| VALINE | -0.015002677 | 8.60E-01 |  |
| XANTHINE | -0.565531775 | 2.73E-01 |  |
| XANTHOSINE | -0.631246327 | 3.36E-01 |  |

Table S 4: Elastic Net regression model (linear relationship): estimated glomerular filtration rate (egfr) ~ Metabolites at BL; 10 fold cv *R^2^* = 0.6233, (simple *R^2^* = 0.8036), N=136, minimal lambda = 2.017 (10 fold-CV), Selected markers 34

| **Variable** | **direction** | **Lambda ratio** |
| --- | --- | --- |
| CREATININE_pos | - | 9.75 |
| GUANIDINOSUCCINATE_neg | - | 6.234 |
| CREATININE_neg | - | 5.685 |
| N-ACETYLNEURAMINATE_pos | - | 5.479 |
| KYNURENATE_pos | - | 5.454 |
| N-ACETYLNEURAMINATE_neg | - | 4.412 |
| GLUCONATE_neg | - | 4.372 |
| N,N-DIMETHYLARGININE_neg | - | 3.52 |
| METHYL JASMONATE_neg | - | 2.954 |
| RIBOSE (ALDOPENTOSES C5H10O5)_pos | - | 2.9 |
| N-FORMYL-L-METHIONINE_neg | - | 2.732 |
| AMINOPHENOL_pos | + | 2.39 |
| TRIGONELLINE_pos | - | 2.39 |
| CYSTINE_pos | - | 2.21 |
| SACCHARATE_neg | - | 2.149 |
| DEOXYCARNITINE_pos | - | 2.11 |
| GLUTAMATE_pos | + | 2.015 |
| ISOLEUCINE_neg | + | 1.96 |
| 2,4-DIHYDROXYACETOPHENONE_neg | - | 1.881 |
| DEOXYGUANOSINE_pos | - | 1.615 |
| GLUTARYLCARNITINE_pos | - | 1.615 |
| 5-METHOXYINDOLEACETATE_neg | - | 1.6 |
| 10-HYDROXYDECANOATE_neg | - | 1.578 |
| GLYCEROPHOSPHOCHOLINE_pos | + | 1.521 |
| TAURINE_neg | - | 1.473 |
| LEUCINE_neg | + | 1.466 |
| 7-METHYLXANTINE_pos | - | 1.331 |
| AMINOISOBUTANOATE_neg | + | 1.277 |
| GLUTAMINE_pos | - | 1.277 |
| N-METHYL-ALANINE_neg | + | 1.259 |
| CREATINE_neg | + | 1.181 |
| GUAIACOL_neg | + | 1.154 |
| BILIVERDIN_pos | - | 1.133 |
| CHENODEOXYCHOLATE_pos | - | 1.023 |

Table S 5: Elastic Net regression model (linear relationship): glucose ~ Metabolites at BL; 10 fold cv *R^2^* = 0.9697, (simple *R^2^* = 0.9841), N=136, minimal lambda = 1.397 (10 fold-CV), Selected markers 32

| **Variable** | **direction** | **Lambda_ratio** |
| --- | --- | --- |
| 3-HYDROXYMETHYLGLUTARATE_pos | + | 52.933 |
| 3-METHYLGLUTACONATE_pos | + | 52.933 |
| ALPHA-D-GLUCOSE (MONOSACCHARIDE C6H12O6)_pos | + | 45.884 |
| L-GULONOLACTONE_neg | + | 25.2 |
| 2-PROPENOATE_neg | + | 24.969 |
| 3-METHYLGLUTACONATE_neg | + | 22.875 |
| ASCORBATE_neg | + | 19.647 |
| OXALOMALATE_pos | - | 9.931 |
| ISOLEUCINE_neg | + | 6.619 |
| SACCHARATE_pos | - | 5.53 |
| PIPECOLATE_pos | - | 3.95 |
| 5-HYDROXYINDOLEACETATE_pos | - | 3.703 |
| URATE_pos | - | 3.424 |
| INOSINE-MONOPHOSPHATE_pos | - | 3.408 |
| TRYPTOPHAN_pos | - | 2.847 |
| METHIONINE SULFOXIMINE_neg | + | 2.669 |
| ASPARAGINE_pos | + | 2.621 |
| NICOTINAMIDE_pos | - | 2.346 |
| GLYCINE_neg | - | 2.303 |
| LAURATE_neg | + | 1.889 |
| L-CARNITINE_pos | - | 1.881 |
| GLUCOSE 1-PHOSPHATE (C6H13O9P)_neg | + | 1.838 |
| CYSTINE_pos | + | 1.747 |
| RIBOSE (ALDOPENTOSES C5H10O5)_pos | - | 1.557 |
| 3-HYDROXYMETHYLGLUTARATE_neg | + | 1.535 |
| N-ACETYLTRYPTOPHAN_pos | - | 1.4 |
| TYROSINE_neg | + | 1.331 |
| METHIONINE_neg | + | 1.231 |
| O-ACETYLCARNITINE_pos | + | 1.154 |
| 4-COUMARATE_neg | + | 1.102 |
| DEOXYHEXOSE_pos | - | 1.087 |
| GLYCOCHENODEOXYCHOLATE_neg | + | 1.019 |

Table S 6: Elastic Net regression model (linear relationship): hba1c ~ Metabolites at BL; 10 fold cv *R^2^* = 0.2749, (simple *R^2^* = 0.5019), N=136, minimal lambda = 0.186 (10 fold-CV), Selected markers 20

| **Variable** | **direction** | **Lambda ratio** |
| --- | --- | --- |
| 3-HYDROXYMETHYLGLUTARATE_pos | + | 3.932 |
| DEOXYHEXOSE_pos | - | 3.842 |
| DEOXYHEXOSE_neg | - | 3.488 |
| 3-METHYLGLUTACONATE_pos | + | 3.377 |
| D-MANNOSAMINE (AMINO SUGAR C6H13NO5)_pos | + | 2.821 |
| GLUCONATE_neg | + | 2.597 |
| 3-METHYLGLUTACONATE_neg | + | 2.22 |
| RAFFINOSE_pos | + | 1.779 |
| ISOLEUCINE_neg | + | 1.763 |
| LUMICHROME_pos | - | 1.699 |
| LACTOSE (DISACCHARIDE C12H22O11)_pos | + | 1.653 |
| TRANS-ACONITATE_pos | - | 1.623 |
| SEROTONINE_pos | + | 1.507 |
| 3-HYDROXYMETHYLGLUTARATE_neg | + | 1.487 |
| 3,4,5-TRIMETHOXYCINNAMATE_pos | - | 1.446 |
| N-ACETYLNEURAMINATE_neg | + | 1.368 |
| ASPARAGINE_pos | + | 1.271 |
| BILIVERDIN_neg | - | 1.175 |
| GLUTARYLCARNITINE_pos | - | 1.159 |
| CITRATE_pos | - | 1.112 |

Table S 7: Elastic Net regression model (linear relationship): fatty liver index (fli) ~ Metabolites at BL; 10 fold cv *R^2^* = 0.4069, (simple *R^2^* = 0.7460), N=136, minimal lambda = 2.778 (10 fold-CV), Selected markers 43

| **Variable** | **direction** | **Lambda ratio** |
| --- | --- | --- |
| GLUTAMATE_pos | + | 7.778 |
| GLUTAMATE_neg | + | 7.159 |
| O-ACETYLSERINE_neg | + | 7.159 |
| GLUTATHIONE REDUCED_pos | - | 6.036 |
| GLUTAMINE_neg | - | 5.137 |
| RAFFINOSE_pos | + | 3.772 |
| KYNURENINE_pos | + | 3.703 |
| URATE_pos | + | 3.602 |
| 3,4,5-TRIMETHOXYCINNAMATE_pos | - | 3.504 |
| HYDROXYPHENYLLACTATE_neg | + | 3.456 |
| ASPARAGINE_neg | - | 3.361 |
| L-CARNITINE_pos | + | 3.181 |
| L-ALANINE_pos | + | 3.137 |
| KYNURENINE_neg | + | 2.9 |
| UROCANATE_pos | - | 2.757 |
| N-METHYLARGININE_pos | + | 2.633 |
| 10-HYDROXYDECANOATE_neg | - | 2.526 |
| PARAXANTHINE_neg | + | 2.514 |
| THEOPHYLLINE_neg | + | 2.514 |
| BETAINE_pos | - | 2.446 |
| CAFFEINE_pos | + | 2.272 |
| SACCHARATE_pos | - | 2.23 |
| PYROCATECHOL_neg | - | 2.21 |
| L-GULONOLACTONE_neg | + | 2.034 |
| INDOLE-3-PYRUVATE_pos | + | 2.034 |
| GAMMA-AMINOBUTYRATE_pos | - | 1.916 |
| OXOGLUTARATE_pos | + | 1.872 |
| CIS-4-HYDROXY-D-PROLINE_pos | + | 1.855 |
| L-ALANINE_neg | + | 1.755 |
| PYRIDOXAMINE_pos | + | 1.653 |
| GLUCOSE 1-PHOSPHATE (C6H13O9P)_pos | + | 1.638 |
| GLYCINE_neg | - | 1.586 |
| BILIVERDIN_pos | - | 1.586 |
| INDOLE-3-ACETATE_pos | - | 1.564 |
| 1-METHYL-L-HISTIDINE_neg | - | 1.5 |
| N-ACETYLPHENYLALANINE_neg | - | 1.487 |
| URATE_neg | + | 1.459 |
| LEUCINE_pos | + | 1.439 |
| METHYL JASMONATE_neg | - | 1.413 |
| ADENOSINE-MONOPHOSPHATE_pos | + | 1.277 |
| PHOSPHOCREATINE_pos | + | 1.191 |
| URIDINE_pos | + | 1.127 |
| O-ACETYLCARNITINE_pos | + | 1.047 |

Table S 8: Elastic Net regression model (linear relationship): fibrosis-4 score (fib4) ~ Metabolites at BL; 10 fold cv *R^2^* = 0.3170, (simple *R^2^* = 0.7419), N=136, minimal lambda = 0.132 (10 fold-CV), Selected markers 35

| **Variable** | **direction** | **Lambda ratio** |
| --- | --- | --- |
| GLYCOCHOLATE_pos | + | 9.977 |
| GLYCOCHENODEOXYCHOLATE_pos | + | 9.795 |
| GLYCOCHOLATE_neg | + | 9.528 |
| GLYCOCHENODEOXYCHOLATE_neg | + | 9.44 |
| CYSTINE_neg | + | 4.024 |
| EMPAGLIFLOZIN_neg | + | 3.878 |
| EMPAGLIFLOZIN_pos | + | 3.703 |
| BILIVERDIN_pos | + | 3.408 |
| GLUTATHIONE REDUCED_pos | - | 3.361 |
| ADENOSINE 3,5-DIPHOSPHATE_neg | - | 3.21 |
| INOSINE-MONOPHOSPHATE_neg | - | 3.065 |
| ISOCITRATE_neg | + | 2.732 |
| DOCOSAHEXAENOATE_pos | + | 2.549 |
| DEOXYCARNITINE_pos | + | 2.48 |
| 5-HYDROXYINDOLEACETATE_pos | + | 2.379 |
| CYSTINE_pos | + | 2.368 |
| XANTHINE_pos | + | 2.261 |
| N-ACETYLSEROTONIN_pos | + | 2.22 |
| TRANS-ACONITATE_pos | + | 2.062 |
| N,N-DIMETHYLARGININE_pos | + | 1.779 |
| 10-HYDROXYDECANOATE_neg | + | 1.771 |
| ISOLEUCINE_neg | - | 1.731 |
| NICOTINAMIDE_pos | - | 1.707 |
| N8-ACETYLSPERMIDINE_pos | + | 1.66 |
| DEOXYURIDINE-MONOPHOSPHATE_pos | - | 1.615 |
| N-FORMYL-L-METHIONINE_neg | + | 1.608 |
| OXOGLUTARATE_neg | + | 1.473 |
| INDOLE-3-PYRUVATE_neg | - | 1.466 |
| DEOXYGUANOSINE_pos | + | 1.466 |
| CYSTEINE_neg | + | 1.4 |
| GUANIDINOSUCCINATE_pos | + | 1.362 |
| GUAIACOL_neg | - | 1.319 |
| 4-COUMARATE_pos | + | 1.242 |
| ASPARTATE_pos | - | 1.242 |
| S-ADENOSYLHOMOCYSTEINE_pos | - | 1.186 |

Table S 9: Elastic Net regression model (linear relationship): fatty liver disease (fld) ~ Metabolites at BL; 5 fold cv AUC= 0.9038, (simple AUC = 0.9994), N=142, minimal lambda = 0.027 (5 fold-CV), Selected markers 55

| **Variable** | **direction** | **Lambda ratio** |
| --- | --- | --- |
| GLUTAMATE_pos | + | 13.525 |
| GLUTAMATE_neg | + | 13.339 |
| O-ACETYLSERINE_neg | + | 13.339 |
| GLUTATHIONE REDUCED_pos | - | 12.68 |
| 10-HYDROXYDECANOATE_neg | - | 8.729 |
| AMINOISOBUTANOATE_neg | + | 8.569 |
| N-METHYL-ALANINE_neg | + | 8.569 |
| KYNURENINE_pos | + | 7.192 |
| L-CARNITINE_pos | + | 6.529 |
| N-METHYLARGININE_pos | + | 6.263 |
| GLYCERATE_neg | + | 6.206 |
| L-ALANINE_pos | + | 6.064 |
| INDOLE-3-PYRUVATE_pos | + | 5.954 |
| 3,4,5-TRIMETHOXYCINNAMATE_pos | - | 5.633 |
| 1-METHYLADENOSINE_pos | + | 5.257 |
| RAFFINOSE_pos | + | 5.233 |
| UROCANATE_pos | - | 4.728 |
| GLUCOSE 1-PHOSPHATE (C6H13O9P)_pos | + | 4.663 |
| PARAXANTHINE_neg | + | 4.432 |
| GLYCINE_neg | - | 4.412 |
| THEOPHYLLINE_neg | + | 4.331 |
| PYROGLUTAMATE_neg | + | 4.005 |
| PARAXANTHINE_pos | - | 3.932 |
| ASPARAGINE_neg | - | 3.86 |
| URATE_neg | + | 3.72 |
| CAFFEINE_pos | + | 3.504 |
| 6-PHOSPHOGLUCONATE_pos | + | 3.393 |
| URATE_pos | + | 3.3 |
| BETAINE_pos | - | 3.27 |
| THYROXINE_neg | - | 3.255 |
| HIPPURATE_pos | - | 3.21 |
| KYNURENINE_neg | + | 3.122 |
| HOMOCYSTEINE_pos | + | 2.847 |
| N-ACETYLNEURAMINATE_pos | + | 2.669 |
| CIS-4-HYDROXY-D-PROLINE_pos | + | 2.514 |
| PHENYLACETALDEHYDE_pos | + | 2.457 |
| THEOBROMINE_pos | + | 2.412 |
| OXOGLUTARATE_pos | + | 2.282 |
| 4-COUMARATE_pos | + | 2.053 |
| CYTIDINE_neg | - | 1.96 |
| AMINOPHENOL_pos | + | 1.907 |
| URIDINE_pos | + | 1.855 |
| 4-COUMARATE_neg | + | 1.821 |
| 2-AMINOISOBUTYRATE_pos | - | 1.821 |
| CORTISONE_pos | + | 1.691 |
| N-ACETYL CARNOSINE_neg | + | 1.66 |
| CYSTEINE_neg | + | 1.615 |
| PYROCATECHOL_neg | - | 1.466 |
| GUANIDINOACETATE_pos | + | 1.301 |
| PANTOTHENATE_pos | + | 1.289 |
| ADENOSINE-MONOPHOSPHATE_pos | + | 1.225 |
| CREATININE_neg | - | 1.17 |
| CHENODEOXYCHOLATE_pos | - | 1.164 |
| PHENYLETHANOLAMINE_pos | + | 1.097 |
| GAMMA-AMINOBUTYRATE_pos | - | 1.033 |

Table S 10: Elastic Net regression model (linear relationship): liver fibrosis (fib4_bin) ~ Metabolites at BL; 5 fold cv AUC= 0.7249, (simple AUC = 0.9987), N=142, minimal lambda = 0.044 (5 fold-CV), Selected markers 57

| **Variable** | **direction** | **Lambda ratio** |
| --- | --- | --- |
| CYSTINE_pos | + | 6.498 |
| KYNURENINE_neg | + | 5.791 |
| CREATININE_neg | + | 4.861 |
| GUANIDINOSUCCINATE_neg | + | 4.861 |
| ADENOSINE-MONOPHOSPHATE_neg | - | 4.642 |
| HYPOXANTHINE_neg | + | 4.494 |
| INOSINE-MONOPHOSPHATE_neg | - | 4.252 |
| ADENOSINE-MONOPHOSPHATE_pos | - | 3.987 |
| HIPPURATE_neg | + | 3.968 |
| SEROTONIN_pos | - | 3.842 |
| CORTISOL_neg | + | 3.686 |
| GLYCEROPHOSPHOCHOLINE_pos | - | 3.619 |
| METHYLMALONATE_neg | + | 3.553 |
| PROLINE_neg | - | 3.504 |
| HYPOXANTHINE_pos | + | 3.488 |
| TRANS-ACONITATE_pos | + | 3.346 |
| CHENODEOXYCHOLATE_pos | + | 3.27 |
| GALACTURONATE_neg | + | 2.996 |
| THEOBROMINE_neg | + | 2.914 |
| TRIMETHYLAMINE N-OXIDE_pos | + | 2.847 |
| GUANIDINOACETATE_pos | - | 2.821 |
| NICOTINAMIDE_pos | - | 2.757 |
| OXALOMALATE_pos | - | 2.412 |
| DOCOSAHEXAENOATE_pos | + | 2.368 |
| CAFFEINE_pos | + | 2.282 |
| CYTIDINE_neg | - | 2.13 |
| N,N,N-TRIMETHYLLYSINE_pos | - | 2.081 |
| THYROXINE_neg | - | 2.015 |
| DEOXYHEXOSE_pos | - | 1.96 |
| N-ACETYLTRYPTOPHAN_pos | + | 1.881 |
| DEOXYURIDINE-MONOPHOSPHATE_pos | - | 1.872 |
| ASCORBATE_neg | + | 1.846 |
| CITRATE_pos | + | 1.821 |
| SACCHARATE_pos | + | 1.747 |
| 5-METHYLCYTIDINE_neg | - | 1.739 |
| GAMMA-AMINOBUTYRATE_pos | - | 1.739 |
| XANTHOSINE_neg | + | 1.699 |
| DEOXYGUANOSINE_pos | + | 1.66 |
| L-CARNITINE_pos | - | 1.66 |
| PALMITOLEATE_pos | - | 1.653 |
| L-ORNITHINE_pos | - | 1.645 |
| TRYPTAMINE_neg | + | 1.608 |
| QUINOLINE_pos | + | 1.6 |
| INDOLE-3-METHYL ACETATE_neg | + | 1.593 |
| GUAIACOL_neg | - | 1.564 |
| THEOBROMINE_pos | + | 1.528 |
| GUANIDINOSUCCINATE_pos | + | 1.453 |
| URIDINE_pos | - | 1.453 |
| GLUTAMINE_pos | + | 1.446 |
| PANTOTHENATE_pos | + | 1.407 |
| GLYCINE_pos | + | 1.381 |
| TRANS-CINNAMATE_pos | - | 1.265 |
| ASPARAGINE_pos | + | 1.231 |
| METHIONINE_pos | + | 1.191 |
| CHOLATE_neg | - | 1.175 |
| GLYCOCHENODEOXYCHOLATE_pos | + | 1.148 |
| HOMOCYSTEINE_pos | - | 1.092 |

Table S 11: Elastic Net regression model (linear relationship): hypertension ~ Metabolites at BL; 5 fold cv AUC= 0.7011, (simple AUC = 0.9231), N=142, minimal lambda = 0.08 (5 fold-CV), Selected markers 14

| **Variable** | **direction** | **Lambda ratio** |
| --- | --- | --- |
| SEROTONIN_pos | - | 2.585 |
| GLUTATHIONE REDUCED_pos | - | 2.457 |
| HIPPURATE_pos | - | 1.872 |
| 1-METHYL-L-HISTIDINE_neg | - | 1.838 |
| N,N,N-TRIMETHYLLYSINE_pos | - | 1.691 |
| OXALOMALATE_pos | - | 1.593 |
| 3,4,5-TRIMETHOXYCINNAMATE_pos | - | 1.446 |
| 5-METHYLCYTIDINE_neg | - | 1.439 |
| ISOCITRATE_pos | - | 1.301 |
| CYSTINE_pos | + | 1.295 |
| THEOBROMINE_neg | - | 1.271 |
| L-CARNITINE_pos | + | 1.225 |
| N-METHYLARGININE_pos | + | 1.122 |
| PROLINE_pos | + | 1.014 |

Table S 12: Elastic Net regression model (linear relationship): obesity (adipositas) ~ Metabolites at BL, 5 fold cv AUC= 0.7307, (simple AUC = 0.8591), N=142, minimal lambda = 0.132 (5 fold-CV), Selected markers 18

| **Variable** | **direction** | **Lambda ratio** |
| --- | --- | --- |
| AMINOISOBUTANOATE_neg | + | 3.137 |
| N-METHYL-ALANINE_neg | + | 3.137 |
| O-ACETYLSERINE_neg | + | 2.996 |
| GLUTAMATE_neg | + | 2.968 |
| GLUTAMATE_pos | + | 2.2 |
| ASPARAGINE_neg | - | 2.091 |
| HYDROXYPHENYLLACTATE_neg | + | 1.889 |
| OXOGLUTARATE_neg | + | 1.473 |
| TYRAMINE_neg | + | 1.453 |
| L-CARNITINE_pos | + | 1.413 |
| GLUTAMINE_neg | - | 1.394 |
| TYROSINE_pos | + | 1.368 |
| 1-METHYLNICOTINAMIDE_pos | - | 1.191 |
| L-ALANINE_pos | + | 1.181 |
| 3-HYDROXYMETHYLGLUTARATE_neg | + | 1.107 |
| 5-METHYLCYTIDINE_neg | - | 1.042 |
| 4-COUMARATE_neg | + | 1.033 |
| KYNURENINE_pos | + | 1.019 |

Table S 13: Elastic Net regression model (linear relationship): chronic kidney disease (cdk) ~ Metabolites at BL; 5 fold cv AUC= 0.6050, (simple AUC = 0.8418), N=139, minimal lambda = 0.108 (5 fold-CV), Selected markers 10

| **Variable** | **direction** | **Lambda ratio** |
| --- | --- | --- |
| INOSINE-MONOPHOSPHATE_neg | + | 2.159 |
| ALPHA-D-GLUCOSE (MONOSACCHARIDE C6H12O6)_pos | - | 1.812 |
| 3-HYDROXYMETHYLGLUTARATE_pos | - | 1.493 |
| L-ALANINE_pos | - | 1.307 |
| GAMMA-LINOLENATE_pos | + | 1.253 |
| TYROSINE_pos | - | 1.197 |
| ADENOSINE-MONOPHOSPHATE_neg | + | 1.186 |
| AMINOPHENOL_pos | - | 1.186 |
| CREATININE_neg | - | 1.019 |
| SARCOSINE_neg | - | 1.014 |

**References**

(1) Kirwan, J. A.; Broadhurst, D. I.; Davidson, R. L.; Viant, M. R. Characterising and correcting batch variation in an automated direct infusion mass spectrometry (DIMS) metabolomics workflow. *Anal Bioanal Chem* **2013**, *405* (15), 5147-5157. DOI: 10.1007/s00216-013-6856-7 From NLM Medline.
